# A novel regulatory circuit of ATG4B and SESN3 promotes T cell leukemogenesis

**DOI:** 10.1186/s13046-025-03588-0

**Published:** 2025-11-22

**Authors:** Wenjuan Ma, Lei Zhang, Haixia Zhou, Xiuyan Zhang, Xingjie Qin,, Yan Wan, Rongyao Ma, Xueyan Song, Xiaonan Zhou, Hong Liu, Bo Hu, Depei Wu, Jianrong Wang, Xiaoyan Jiang, Yun Zhao

**Affiliations:** 1 Cyrus Tang Medical Institute, National Clinical Research Center for Hematologic Diseases, Collaborative Innovation Center of Hematology, Soochow University, Suzhou, 215123 China; 2 The First Affiliated Hospital of Soochow University, Jiangsu Institute of Hematology, Suzhou, 215006 China; 3 Institute of Blood and Marrow Transplantation, Soochow University, Suzhou, 215123 China; 4 Suzhou Ninth Hospital Affiliated to Soochow University, Suzhou, 215200 China; 5 British Columbia Cancer Research Institute and Department of Medical Genetics, Terry Fox Laboratory, University of British Columbia, BC Vancouver, V5Z 1L3 Canada; 6 NHC Key Laboratory of Thrombosis and Hemostasis, MOE Engineering Center of Hematological Disease, Soochow University, Suzhou, 215006 China

**Keywords:** ATG4B, SESN3, T-ALL, ATG4B inhibitor

## Abstract

**Background:**

T cell acute lymphoblastic leukemia is a fatal hematological malignancy. Despite the treatment progress, no targeted therapy is available currently, which urges to deepen the understanding of the underlying mechanism of T-ALL cell growth/survival. Autophagy is a conserved cellular process, which plays a dual role in human cancers. Nevertheless, many aspects of the involvement of autophagy in T-ALL are not fully understood.

**Methods:**

T-ALL patient cells and normal control cells were subjected to RT‒qPCR analysis. Gene silence and overexpression was used to study the function of ATG4B and sestrin 3 (SESN3) in T-ALL cells. Atg4b deficient mice were used to study the role of Atg4b in normal hematopoietic cells and T cell development. The efficacy of S130, an ATG4B inhibitor to suppress T-ALL cell growth was evaluated in xenograft models.

**Results:**

The results showed that the expression of several autophagy-related genes (especially *ATG4B*) was significantly higher in T-ALL patient cells than control cells. ATG4B ablation decreased autophagic flux and inhibited T-ALL cell growth. In contrast, Atg4b depletion had mild effects on normal hematopoiesis and T cell development. RNA-seq data and subsequent studies revealed a novel regulatory circuit of ATG4B and SESN3, and the results indicated that SESN3 hampered T-ALL cell growth via the inhibition of both mTOR/S6K/protein synthesis pathway and autophagy. Importantly, S130 exhibited anti-leukemia activity in xenograft models.

**Conclusions:**

The present study demonstrates that a novel ATG4B-SESN3 regulatory circuit plays a crucial role in T cell leukemogenesis, which suggests that targeting ATG4B is a promising strategy for T-ALL treatment.

**Supplementary Information:**

The online version contains supplementary material available at 10.1186/s13046-025-03588-0.

## Introduction

T cell acute lymphoblastic leukemia (T-ALL) is a lethal hematological malignancy characterized by the abnormal growth of immature thymocytes in both the thymus and bone marrow [1, 2], which accounts for approximately 15% of pediatric ALL cases and 25% of adult ALL cases [3]. Despite improvements in intensive chemotherapy regimens, drug resistance or early relapse is still a big challenge for some patients, who usually have a dismal prognosis [4–6]. The progress of T-ALL treatment is relatively slow compared with other types of leukemias, as no effective targeted therapy for T-ALL is available and no cellular immunotherapy or immune-agent has been licensed yet [7, 8]. Therefore, it is urgent to obtain deeper molecular insights into the growth and survival mechanisms of T-ALL cells, which might lead to novel therapeutic strategies.

Autophagy is a conserved adaptive process for cell survival under conditions of nutrient deprivation or caloric restriction, in which cytoplasmic components are degraded or recycled via a lysosome-mediated pathway to maintain metabolic homoeostasis [9]. Although autophagy plays a critical role in multiple physiological processes and human diseases [10], its role in normal hematopoiesis and hematological malignancies, particularly in T-ALL has not been fully explored [11–13]. Genetic defect of autophagy-related gene 7 (Atg7) leads to a lethal pre-leukemic phenotype in mice [14]. In line with this, Atg5 loss accelerates MLL-ENL induced leukemia in a mice model [15]. These findings indicate that proper autophagy activities prevent the initiation of hematological malignancies. On the contrary, ATG4B is identified as a potential biomarker and therapeutic target of tyrosine kinase inhibitor-resistant chronic myeloid leukemia (CML) stem/progenitor cells [16]. ATG7 is highly expressed in human CML cell, and gene silencing of ATG7 results in growth inhibition of leukemic cells [17]. Similarly, Atg5/7 is required for the maintenance of leukemia-initiating cells in murine myeloid leukemia [18]. These reports suggest an indispensable role of autophagy in leukemia progression. Meanwhile, the administration of autophagy inhibitors hampers T-ALL cell growth [19, 20] and some compounds impair T-ALL cell proliferation through autophagy signaling [21, 22], which highly suggests that autophagy plays an important role in T-ALL progression. However, the direct study on the roles of autophagy players in T-ALL is still lacking.

ATG4 plays a critical role in autophagosome formation by cleaving full-length microtubule-associated protein 1A/1B-light chain 3 (pro-LC3) into LC3-I to facilitate the conjugation of phosphatidylethanolamine (PE) and the generation of membrane-bound LC3-II. In addition, membrane-localized LC3-II requires ATG4 to remove PE for LC3-I recycling. In mammals, there are four ATG4 paralogs (ATG4A, ATG4B, ATG4C, and ATG4D). Among these, ATG4B is the most catalytically active [23, 24]. Interestingly, a previous study showed that Atg4b knockout mice were viable and appeared normal in a battery of screening tests [25], which supports the idea that ATG4B is an attractive target for cancer treatment. Due to the fact that ATG4B expression is elevated in various cancers and promotes cancer cell growth [23, 26–28], ATG4B inhibitors have been developed and investigated in pre-clinical studies [29, 30]. However, the anti-leukemia activity of ATG4B inhibitor has not been evaluated.

The sestrin family consists of several conserved stress-inducible metabolic proteins, namely, SESN1–3 [31]. These proteins protect cells against various stimuli, mainly through the activation of AMP-dependent protein kinase (AMPK) and the inhibition of mammalian target of rapamycin complex 1 (mTORC1) [32, 33]. SESN3 is a poorly studied member in this protein family. It is proposed that SESN2 plays a dual role in human cancers [34]. Previous studies showed that SESN3 inhibited CML cell growth and its recurrent missense mutations were identified in benzene-induced pediatric leukemia [35, 36]. Nevertheless, the role of SESN3 in T-ALL is entirely unknown.

In the present study, the differential expression of several autophagy-related genes in T-ALL patients sample compared with normal cells, especially *ATG4B* was reported. The role of ATG4B in T-cell leukemogenesis and T cell development was investigated in multiple cellular and mice models. Mechanistically, a mutually regulated circuit between ATG4B and SESN3 was identified to promote T-cell leukemogenesis. The efficacy of S130, an ATG4B inhibitor to hamper T-ALL cell growth was evaluated as well. Our data demonstrate that ATG4B presents a new vulnerability of T-ALL cells, which suggests that targeting ATG4B provides a promising strategy for T-ALL treatment.

## Methods

### Cell lines and primary cells

Jurkat (no. SCSP-513), MOLT-4 (no. TCHu224), CCRF-CEM (no. TCHu147), and 293 T (no. SCSP-502) cells were purchased from the National Collection of Authenticated Cell Cultures (www.cellbank.org.cn, Shanghai, China). The cell lines were confirmed to be free of mycoplasma contamination, and their identities were authenticated by short tandem repeat (STR) profiling. As described in our previous studies [37, 38], bone marrow cells (BMCs) from patients with T-ALL and NBM cells from healthy donors were separated using gradient centrifuge with Lympholyte-H cell separation media (Cedarlane Laboratories, Burlington, NC, USA) to obtain nucleated cells, which were further processed with EasySep kit (STEMCELL Technologies, Vancouver, Canada) to enrich CD3^+^ or CD34^+^ cells. The clinical information of T-ALL patients are shown in Supplemental Table S1. The clinical samples were obtained in accordance with the Declaration of Helsinki, with the approved standards of the Ethical Committee of Soochow University (Suzhou, China).

### Monodansylcadaverine (MDC) staining

MDC staining assay was performed using a staining kit (C3018M, Beyotime, Shanghai, China) following the manufacturer’s instruction. In brief, cells were first collected and washed with PBS and then resuspended in Earle's Balanced Salt Solution (EBSS) (1 × 10^6^ cells/mL). After incubation at 37 °C for 2 h, the cells were stained with MDC at 37 °C in the dark for 30 min. Lastly, the cells were washed three times with Assay Buffer and then analyzed by flow cytometry (Gallios, Beckman Coulter, Indianapolis, Indiana, United States).

### RNA preparation and reverse transcription quantitative PCR (RT‒qPCR)

RNA extract and gene expression measurement were conducted as previously described [37]. The primers to detect individual genes are listed Supplemental Table S2. *β-ACTIN* served as the internal control for RT‒qPCR analysis.

### Western blotting

Protein samples were prepared with lysis buffer (Beyotime), and protein samples with equal amount were separated via SDS-PAGE and then transferred onto a PVDF membrane (Millipore, Billerica, MA, USA). The details of antibodies are summarized in Supplemental Table S3. An enhanced chemiluminescence (ECL) detection agent (New Cell & Molecular Biotech, Suzhou, China) was used to obtain the signal.

### Analysis mCherry-GFP-LC3B

Modified from a previous report [16], Jurkat cells were transduced by a retroviral vector containing mCherry-GFP-LC3B, then they were treated by an ATG4B inhibitor and vehicle. Autophagy was analyzed by a confocal microscope (FV1000MPE-share; Olympus, Tokyo, Japan).

### Lentiviral vectors and production

Small-hairpin sequences against ATG4B and SESN3, together with the scrambled control sequence (Supplemental Table S4) were subcloned into lentiviral vectors by GenePharma Co., Ltd. (Shanghai, China). These primers (forward, 5ʹ- CGGGATCCGCCACCATGAACCGGGGCGGCGGCAGCCCG-3ʹ; reverse, 5ʹ CGGGATCCTCAGGTCAAATGCCGAGTTATGGCACG-3ʹ) were used to subclone SESN3 (NM_144665.4) cDNA into a lentiviral vector. Following previous description [37], lentiviruses were produced. Normal CD3^+^ cells, T-ALL patient cells, and normal CD34^+^cells were cultured and transduced as previously described [37–39].

### CRISPR/Cas9-mediated gene targeting

Based on a previous study [39], ATG4B was targeted using the CRISPR/Cas9 strategy. Briefly, Lenti-Cas9-Blast (Addgene, #52962) was used to deliver Cas9 into Jurkat cells following blasticidin selection (10 μg/mL, Gibco, A11139-03). The sgRNA sequences were designed and subcloned into the Lenti-guided puro-IRES-GFP vector, which was subsequently delivered to Jurkat/Cas9 cells via lentiviral transduction. Transduced cells (GFP^+^) were isolated by FACS. The sequences of the sgRNAs targeting ATG4B and the control (sgNC) are listed in Supplemental Table S5.

### Animals

The female immunodeficient mice (NOD.CB17-*Prkdc*^*scid*^*Il2rg*^*tm1*^/Bcgen, Biocytogen, Beijing, China) aged six to eight weeks were used to generate xenograft models with Jurkat cells or PDX cells as previously described [38]. *Atg4b*^−/−^ mice (generated from C57BL/6N strain) were obtained from GemPharmatech (Nanjing, China) and C57BL/6N mice were used as wild-type (WT) control. For genotyping analysis, genomic tail DNA was collected and analyzed with specific primers (Supplemental Table S6). Flow cytometry was used to analyze cells from the bone marrow, peripheral blood, and spleen of the *Atg4b*^−/−^ and wild type (WT) mice, and the coefficients (ratios of organ weight to body weight) of various organs in *Atg4b*^−/−^ and WT mice were compared. CD3^+^cells from mice were enriched by an immunomaganetic method, and then activated by CD3/CD28 exposure, the cell yield and Ki67 expression were analyzed as a previous report [40]. All the studies were performed in accordance with an institutional protocol approved by the Ethics Committee of Soochow University (Suzhou, China).

### Flow cytometry analysis

The cells from the bone marrow, spleen, peripheral blood, and thymus of the mice were obtained in 2% fetal bovine serum (FBS)-supplemented Hank’s Balanced Salt Solution (called HF afterwards). CD16/32 (223,142, Becton Dickinson) was used as blocking agent to analyze these cells resuspended in 2% HF. A variety of antibodies from eBiosceince/Thermo Fisher Scientific (Waltham, MA, USA), including anti-Ter-119 (17–5921-82), anti-Gr1 (45–5931-80), anti-Mac1 (11–0112-82), anti-B220 (25–0452-82), anti-CD4 (11–0041–82), and anti-CD8 (12–0081–82) were used in the assays by a FACS machine (Beckman, Gallios).

### RNA-seq analysis

RNA samples were collected from three pairs of ATG4B-silenced and control Jurkat cells to generate RNA-seq data (eBGI Genomics, Shenzhen, China). Differentially expressed transcripts were determined based on *p*-values (< 0.05) and fold-changes (> 2).

### Compounds and cell viability assay

An ATG4B inhibitor, S130 was purchased from MedChemExpress LLC (Shanghai, China). The compound was dissolved to make the stock solutions following the manufacturer’s instruction. Briefly, 1 × 10^4^ bone marrow cells from patients with T-ALL or CD3^+^ cells from healthy donors were seeded into 96-well plate and were treated with various concentrations of S130, 24 h later the cell viability was measured using a cell counting kit-8 (CCK-8) (New Cell & Molecular Biotech) following the manufacturer’s instruction. The IC50 values were estimated using nonlinear regression analysis with GraphPad Prism software.

### Protein translation assay

Based on a previous report [41], the protein translation was assayed as follows: 30 min after the addition of puromycin (1 μM) to the culture medium, the cells were collected for Western blotting using an α-puromycin antibody (detailed in Supplemental Table S3). An internal control was used to normalize the loading of each sample, and the intensity of the blot signals represented the protein translation of each sample.

### Statistical analysis

All values are represented as the mean ± SEM from more than 3 biological replicates, and the statistical analysis was performed with Student’s *t* test, in which a *p* value less than 0.05 was considered to indicate a significant difference. The Kaplan‒Meier method was used to assess survival, and the *p* value was estimated using the log-rank test.

## Results

### T-ALL cells have higher MDC staining and elevated expression of several autophagy-related genes

**Fig.1:**
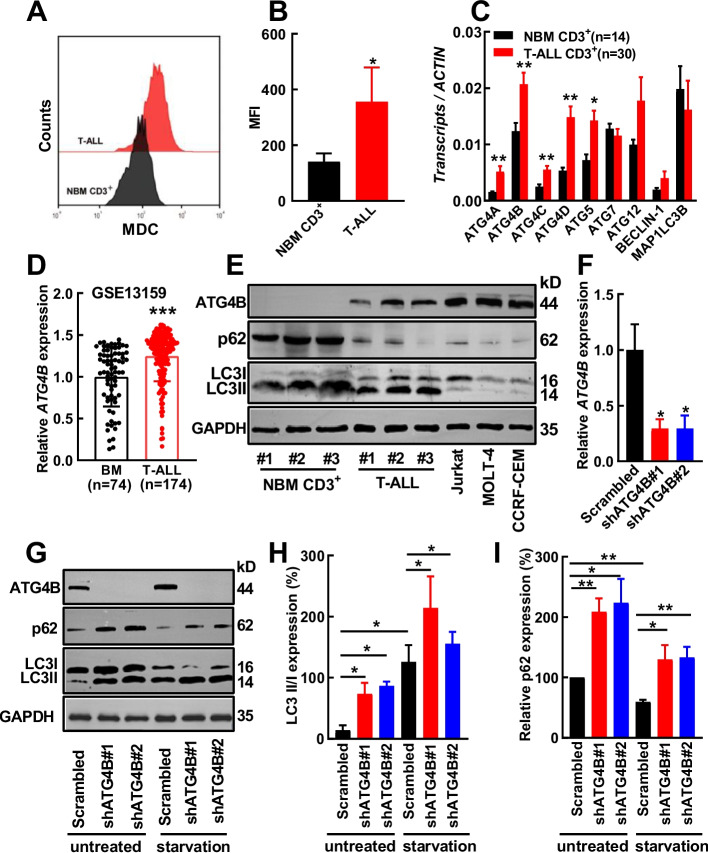
ATG4B is highly expressed in T-ALL patient cells. **A**,** B** Bone marrow cells form patients with T-ALL (*n* = 5) and CD3^+^ cells from the normal bone marrow (NBM) of healthy donors (*n* = 5) were stained with monodansylcadaverine (MDC) (**A**), and the mean fluorescence intensity (MFI) of these cells was compared (**B**). **C** The expression of nine key autophagy genes in CD3^+^ cells from the bone marrow of patients with T-ALL (*n* = 30) and NBM CD3^+^ cells from healthy donors (*n* = 14) was measured by RT‒qPCR and normalized to that of *β-ACTIN*. **D** The expression of *ATG4B* in GSE13159 was analyzed. **E** Western blotting was performed to analyze the expression of ATG4B, p62, and LC3 in NBM CD3.^+^ cells (*n* = 3), BM cells from patients with T-ALL (*n* = 3), and three T-ALL cell lines. GAPDH was used as a loading control. **F** Two shRNA sequences against ATG4B were delivered to Jurkat cells together with the control (Scrambled), then *ATG4B* expression was analyzed by RT‒qPCR in these transduced cells. **G**–**I** ATG4B-silenced and control Jurkat cells were treated with serum-deprivation, four hours later the treated (starvation) cells and control (untreated) cells were subjected to Western blotting analysis (**G**), the expression of LC3II/I (**H**) and p62 (**I**) was quantified. The data are presented as the means ± SEM. Student’s *t* test was used to estimate the statistical significance. *p < 0.05, **p < 0.01, ***p < 0.001

As the role of autophagy in T-ALL progression is not fully understood, the bone marrow (BM) cells from patients with T-ALL and CD3^+^ cells from normal bone marrow (NBM) of healthy donors were stained with monodansylcadaverine (MDC), and the results showed that leukemic cells had significantly higher MDC staining than control cells (Fig. 1A, 1B), suggesting that leukemic cells had higher autophagy activity than control cells. Next, the expression of some autophagy-related genes in CD3^+^ cells from the bone marrow of patients with T-ALL (*n* = 30, Supplementary Table S1) and NBM CD3^+^ cells (*n* = 14) was subjected to RT‒qPCR analysis. Interestingly, all *ATG4* members and *ATG5* had significantly higher expression in T-ALL patient cells than that in control cells (Fig. 1C). Importantly, *ATG4B* had the highest expression among all the tested genes. The highest expression of *ATG4B* among all *ATG4* family members was supported by meta-analysis (Supplementary Fig. S1) and a previous work [16]. Coincidentally, ATG4B has the highest protease activity among all ATG4 members [24]; therefore, subsequent studies focused on ATG4B. The increased expression of *ATG4B* was supported by data from Gene Expression Omnibus (GEO) (GSE13159, Fig. 1D). Additionally, ATG4B protein expression was greater and p62 expression was reduced in primary T-ALL cells and several T-ALL cell lines than NBM CD3^+^ cells (Fig. 1E), which indicated that T-ALL cells had higher autophagy activity than control cells. The level of LC3II/I was negatively correlated with ATG4B expression, suggesting that ATG4B facilitated the removal of PE from LC3II for LC3I recycling in these human cells.

To study how ATG4B regulates autophagy in T-ALL cells, two independent shRNAs were delivered to Jurkat cells together with a control (a scrambled sequence). Both shATG4B#1 and shATG4B#2 effectively inhibited the transcript and protein expression of ATG4B (Fig. 1F, 1G). ATG4B-silenced and control Jurkat cells were cultured with conventional medium (untreated) or serum-deprived medium (starvation), then these cells were analyzed by Western blotting (Fig. 1G‒I), the results showed that ATG4B silencing significantly increased p62, which suggested that autophagic flux was inhibited. The blockade of autophagic flux upon ATG4B silencing occurred even under starvation condition. The accumulation of LC3II upon ATG4B silencing likely suggested its important role in the recycle of LC3I. Additionally, Jurkat cells were transduced by an mCherry-GFP-LC3B vector, and the confocal analysis showed that S130 [30], a specific ATG4B inhibitor strongly inhibited autophagy (Supplementary Fig. S2). Overall, targeting ATG4B was sufficient to inhibit autophagy in T-ALL cells.

### ATG4B ablation inhibits T-ALL cell growth

**Fig.2:**
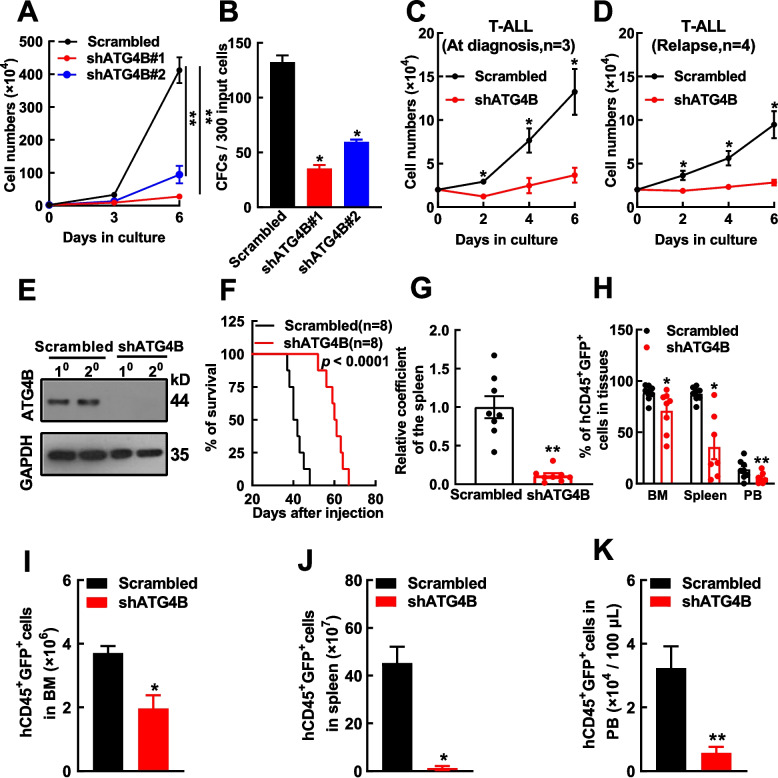
ATG4B is required for T-ALL cell growth. **A**,** B** ATG4B-silenced (shATG4B#1 and #2) and control (Scrambled) Jurkat cells were subjected to cell growth (*n* = 4) (**A**) and colony-forming cell (CFC) (*n* = 4) (**B**) analyses. **C**,** D** ATG4B-silenced and control bone marrow cells from patients with T-ALL at diagnosis (*n* = 3) (**C**) and those in relapse (*n* = 4) (**D**) were subjected to cell growth analysis. **E** ATG4B-silenced and control Jurkat cells were injected into immunodeficient mice through the tail vein (8 × 10^6^ cells/mouse and eight mice in each group). Leukemic cells (hCD45^+^GFP.^+^) from two individual mice in control and shATG4B groups were analyzed for ATG4B expression by Western blotting. **F** The Kaplan‒Meier method was used to assess the survival, and the *p* value was estimated using the log-rank test. **G** Relative coefficients of the spleen (ratios of spleen weight to body weight) in shATG4B and control groups were compared. **H**–**K** The infiltration of leukemic cells in the bone marrow (BM), spleen, and peripheral blood (PB) of these two groups of mice were analyzed by flow cytometry, the percentages of leukemic cells were summarized statistically (**H**), and the absolute numbers of leukemic cells in the BM (**I**), spleen (**J**), and PB (**K**) from shATG4B and control groups are displayed. The data are presented as the means ± SEM. Student’s *t* test was used to estimate the statistical significance. *p < 0.05, **p < 0.01

To investigate the functional role of ATG4B in T-ALL progression, both in vitro and in vivo studies were performed. First, ATG4B silencing significantly inhibited the growth and colony-forming cell (CFC) production in Jurkat cells (Fig. 2A, 2B). Similar results were obtained in MOLT-4 cells (Supplementary Fig. S3A‒E). CRISPR/Cas9 mediated ATG4B depletion led to significant inhibition of cell growth and CFC production in Jurkat cells (Supplementary Fig. S4). Next, ATG4B silencing significant inhibited the growth of leukemic cells from patients with T-ALL, both at diagnosis and in relapse (Figs. 2C, 2D). ATG4B-silenced (shATG4B) and control (Scrambled) Jurkat cells were intravenously (IV) injected into immunodeficient mice through the tail vein. Western blotting showed that ATG4B expression was strongly inhibited in leukemic cells (hCD45^+^GFP^+^) from the ATG4B-silenced group (Fig. 2E). Interestingly, ATG4B silencing significantly prolonged the survival of leukemic mice in comparison to control mice (59 vs. 42 days, *p* < 0.0001, Fig. 2F). Diseased mice were dissected near death and, ATG4B silencing significantly decreased the coefficient of the spleen (ratio of spleen weight to body weight) (75% reduction, *p* < 0.01, Fig. 2G). The infiltration of leukemic cells in the BM, spleen, and peripheral blood (PB) was significantly lower in the ATG4B-silenced group than control (Fig. 2H) as analyzed by flow cytometry (Supplementary Fig. S5). Additionally, the absolute numbers of leukemic cells in the BM, spleen, and PB of ATG4B-silenced group were significantly lower than control group (Fig. 2I‒K). Lastly, the in vivo study using MOLT-4 cells was also performed, and similar results were obtained (Supplementary Fig. S3F‒M).

### ATG4B silencing increases SESN3 expression in T-ALL cells

**Fig.3:**
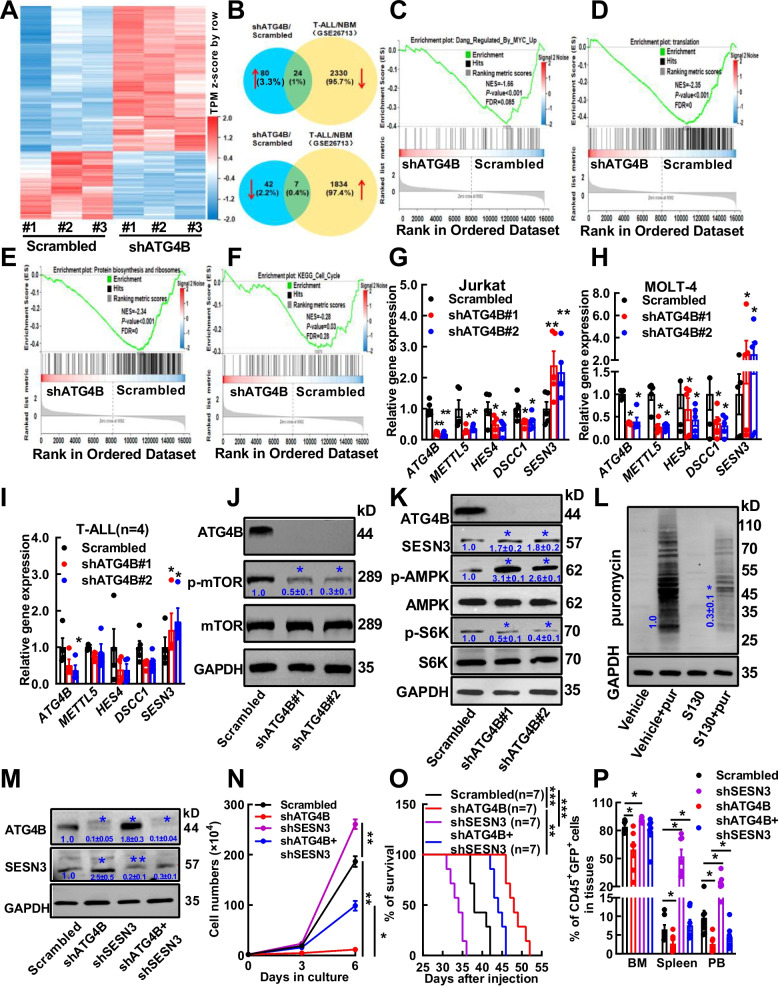
ATG4B silencing requires SESN3 enhancement to suppress T-ALL cell growth. **A** The differentially expressed transcripts between ATG4B-silenced (shATG4B) and control (Scrambled) Jurkat cells revealed by RNA-seq data are displayed in a heatmap. A total of 104 transcripts were upregulated upon ATG4B silencing, while 49 transcripts were downregulated (∣log2FC∣ ≥ 0.5 and *p* < 0.05). **B** Venn diagrams showed that 31 ATG4B-regulated transcripts were also differentially expressed in a public database (GSE26713), in which the gene expression of 117 pediatric T-ALL samples was compared with that of seven normal bone marrow (NBM) controls and the differentially expressed transcripts were identified according to consistent criteria (∣log2FC∣ ≥ 0.5 and *p* < 0.05). **C**–**F** Gene Set Enrichment Analysis was performed to analyze the RNA-seq data. Several pathways were highlighted, including MYC pathway (**C**), translation (**D**), protein biosynthesis and ribosomes (**E**), and cell-cycle (**F**). **G**‒**I** RT–qPCR was used to measure the expression of *ATG4B*, methyltransferase 5 (*METTL5*), hes family bHLH transcription factor 4 (*HES4*), DNA replication and sister chromatid cohesion 1 (*DSCC1*), and sestrin 3 (*SESN3*) in ATG4B-silenced and control Jurkat cells (*n* = 5, G), MOLT-4 cells (*n* = 5, H), and bone marrow cells from patients with T-ALL (*n* = 4, I). **J**,** K** The effects of ATG4B silencing on mTOR (**J**), AMPK, and S6K (**K**) signaling were analyzed by Western blotting. **L** The effect of S130 (an ATG4B inhibitor) on protein synthesis in Jurkat cells was analyzed by puromycin incorporation method. **M**,** N** A “rescue” experiment was performed, the expression of SESN3 and ATG4B in variously transduced cells was analyzed by Western blotting (**M**), and then the growth (**N**) of these cells were measured. **O**,** P** These cells were also injected into immunodeficient mice through the tail vein (8 × 10.^6^ cells/mouse and seven mice in each group). The Kaplan‒Meier method was used to assess the survival, and the *p* value was estimated using the log-rank test (**O**). The infiltration of leukemic cells in the bone marrow (BM), spleen, and peripheral blood (PB) of these groups of mice were analyzed by flow cytometry and summarized statistically (**P**). The data are presented as the means ± SEM, and the statistical significance of the differences was estimated with Student’s *t* test. *p < 0.05; **p < 0.01

To investigate the molecular mechanisms by which ATG4B silencing hampers T-ALL cell growth, RNA-Seq data were generated with ATG4B-silenced and control Jurkat cells from three independent RNA extracts. A total of 104 transcripts were upregulated upon ATG4B silencing, while 49 transcripts were downregulated (∣log2FC∣ ≥ 0.5 and *p* value < 0.05, Fig. 3A, Supplementary Table S7). We then compared our dataset with a public database that identified differentially expressed transcripts by comparing 117 pediatric T-ALL patient samples with seven NBM controls (GSE26713) [42]. The Venn diagram shows that 24 transcripts were upregulated in ATG4B-silenced cells while downregulated in T-ALL samples in comparison to NBM cells; conversely, seven transcripts were downregulated in ATG4B-silenced cells while upregulated in T-ALL samples (Fig. 3B), suggesting that these differentially expressed transcripts may play a role in human T-ALL pathology. Next, Gene set enrichment analysis (GSEA) highlighted that several key pathways were perturbed by ATG4B silencing, including MYC, translation, protein biosynthesis, and the cell cycle (Fig. 3C‒F, Supplementary Table S8). Four candidates were selected for validation based on the known functions of oncogenes or tumor-suppressors, including methyltransferase 5 (*METTL5*), hes family bHLH transcription factor 4 (*HES4*), DNA replication and sister chromatid cohesion 1 (*DSCC1*), and sestrin 3 (*SESN3*) [35, 36, 43–46]. The results showed that *METTL5*, *HES4*, and *DSCC1* were significantly down-regulated upon ATG4B silencing, while *SESN3* was significantly up-regulated upon ATG4B silencing in both Jurkat and MOLT-4 cells (Fig. 3G, 3H). Using T-ALL patient cells for validation, it was found that only *SESN3* was significantly differentially expressed (upregulated) upon ATG4B silencing (*n* = 4, Fig. 3I), but not the other candidate transcripts. As SESN3 is an inhibitor of mTOR signaling [32, 33] and TORC signaling perturbation was suggested by KEGG analysis (Supplementary Fig. S6), mTOR signaling was analyzed by Western blotting. The results showed that mTOR was significantly suppressed (Fig. 3J) in ATG4B-silenced cells, additionally SESN3 expression was increased, AMPK was activated while S6K was inactivated (Fig. 3K). The decreased mTOR/S6K signaling upon ATG4B silencing highly suggested the impaired protein synthesis in ATG4B-silenced cell. Due to the presence of puromycin resistance gene in lentiviral vector to deliver shRNA in this study, we chose to use S130, an ATG4B inhibitor to impair its activity. Subsequently, the puromycin incorporation assay was performed, and which indicated that the protein synthesis in S130-treated cells was significantly decreased than control (Fig. 3L). Rescue experiment was performed to show that additional SESN3 silencing partially reversed ATG4B silencing induced growth inhibition and CFC reduction (Fig. 3M, 3N; Supplementary Fig. S7A). Importantly, the animal model study showed that SESN3 silencing significantly shortened the disease onset of ATG4B-silenced Jurkat cells (Fig. 3O, P; Supplementary Fig. S7C‒G). Moreover, SESN3 silencing conferred Jurkat cells S130 resistance (Supplementary Fig. S7B). Taken together, RNA-seq data and subsequent studies indicated that ATG4B silencing inhibited T-ALL cell growth partially through SESN3 enhancement.

### Autophagy activity regulates SESN3 expression

**Fig.4:**
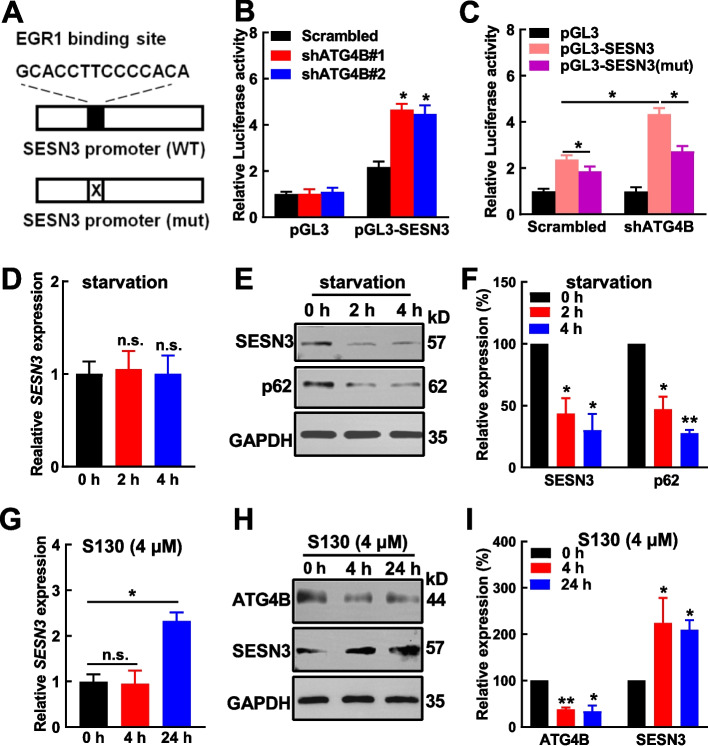
SESN3 expression is regulated by autophagy activity in T-ALL cells. **A** The schematic graph of the structure of wild-type SESN3 promoter (WT) and EGR1-binding site deleted SESN3 promoter (mut) is shown. **B** The activity of SESN3 promoter was assessed in ATG4B-silenced (shATG4B#1 and #2) and control (Scrambled) Jurkat cells. **C** The activities of SESN3 promoter (WT) and its mutant were measured in ATG4B-silenced and control cells. **D**–**F** Jurkat cells were deprived of serum for starvation treatment. The expression of SESN3 was measured by RT–qPCR (**D**) and Western blotting (**E**, **F**). **G**–**I** Jurkat cells were treated with S130, an ATG4B inhibitor for 4 h and 24 h. The expression of SESN3 was measured by RT–qPCR (**G**) and Western blotting (**H**, **I**). The data are presented as the means ± SEM, and the statistical significance of the differences was estimated with Student’s *t* test. *p < 0.05; **p < 0.01; n.s., not significant

To study how SESN3 expression is enhanced by ATG4B silencing, the transcriptional factors were browsed in RNA-seq data. EGR1 and others were up-regulated upon ATG4B silencing (Supplementary Fig. S8A‒C). Further in silico analyses showed that EGR1 had higher regulatory potential than the other candidates (Supplementary Fig. S8D) and the expression of *SESN3* was significantly correlated with that of *EGR1* but not the other candidates (Supplementary Fig. S8E‒G). RT‒qPCR validation showed that *EGR1* expression was significantly upregulated upon ATG4B silencing in Jurkat cells (Supplementary Fig. S8H). These data led to the hypothesis that EGR1 might play a critical role in SESN3 regulation. The reporter constructs of wild-type (WT) and EGR1-binding deficient (mut) SESN3 promoter were generated (Fig. 4A). ATG4B silencing significantly increased SESN3 promoter activity in a reporter assay (Fig. 4B). However, EGR1-binding deficiency almost entirely removed ATG4B silencing-induced reporter activity enhancement (Fig. 4C). Conversely, ATG4B overexpression significantly suppressed the expression of *EGR1* and *SESN3* (Supplementary Fig. S8I‒K), and reduced the reporter activity of SESN3 (WT) promoter but not the mutant one (Supplementary Fig. S8L). These data indicated an important role of EGR1 in the transcriptional regulation of SESN3.

Next, we sought to study whether autophagy activity regulates SESN3 expression. After Jurkat cells were starved by serum-deprivation for 2 h and 4 h, the transcript and protein expression of SESN3 were measured. Our data showed that starvation did not alter transcript expression of *SESN3* (Fig. 4D), while the treatment enhanced autophagy flux as expected (indicated by decreased p62) and significantly decreased protein expression of SESN3 (Fig. 4E, 4F). The reduction of p62 four hours after serum-deprivation was as high as 72% with a greater extent than that observed previously (41% reduction, Fig. 1G, I), which was possibly due to the lentivival transduction in the previous experimental setting. In contrast, Jurkat cells were treated with S130 for 4 h and 24 h. RT‒qPCR showed *SESN3* expression did not change after 4 h-treatment, however significantly increased after 24 h-treatment (Fig. 4G). Western blotting analysis showed that both 4 h- and 24 h-S130 exposure significantly enhanced SESN3 expression (Fig. 4H, 4I). These data indicated that autophagy activity regulates SESN3 expression in T-ALL cells. Overall, we proposed that immediate change of autophagy activity altered protein expression of SESN3; meanwhile, lentiviral vector-delivered shATG4B (typically 3–4 days) or longer exposure of S130 (24 h) triggered EGR1-mediated transcriptional activation of *SESN3*.

### SESN3 plays a novel tumor-suppressor role in T-ALL through the dual inhibition of mTOR signaling and autophagy activity

**Fig.5:**
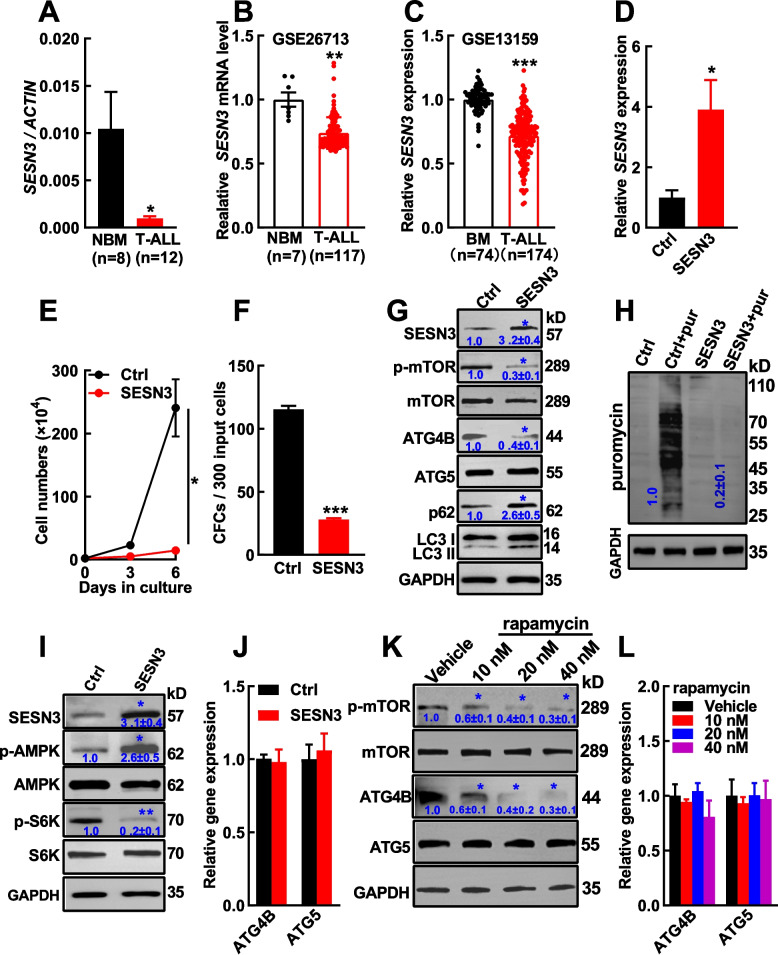
SESN3 inhibits T-ALL cell growth through the dual inhibition of mTORC1/S6K signaling and autophagy. **A** *SESN3* expression was measured in eight normal bone marrow (NBM) CD3^+^ cells and twelve BM cells from patients with T-ALL by RT‒qPCR. **B**,**C** *SESN3* expression was analyzed in GSE26713 (**B**) and GSE13159 (**C**). **D** A lentiviral vector was generated to deliver SESN3 (NM_144665.4, encoding the longer form of SESN3) to Jurkat cells. The transcript expression of *SESN3* in overexpressed- and control Jurkat cells was detected. **E**,** F** The effects of SESN3 overexpression on the growth (**E**) and colony-forming cell (CFC) production (**F**) were analyzed. **G**‒**I** The effects of SESN3 overexpression on mTOR, ATG4B, ATG5, p62, and LC3 were analyzed by Western blotting (**G**). The effects of SESN3 overexpression on protein synthesis (**H**), AMPK, and S6K signaling (**I**) were analyzed. **J** The transcript expression of *ATG4B* and *ATG5* upon SESN3 overexpression was analyzed by RT‒qPCR. **K** Western blotting was used to analyze rapamycin-treated Jurkat cells and their control for the expression of mTOR, ATG4B, and ATG5. **L** The transcript expression of *ATG4B* and *ATG5* upon rapamycin treatment was analyzed by RT‒qPCR. The data are presented as the means ± SEM, and the statistical significance of the differences was estimated with Student’s *t* test. *p < 0.05; **p < 0.01; ***p < 0.001

To explore the role of SESN3 in T-ALL, *SESN3* expression was firstly measure by RT‒qPCR, and the results showed that *SESN3* expression was significantly lower in T-ALL patient samples (n = 12) than control cells (n = 8) (*p* < 0.05, Fig. 5A), which was supported by data from GSE26713 (*p* < 0.01, Fig. 5B) and GSE13159 (*p* < 0.001, Fig. 5C). Later, in gain-of-function studies, RT‒qPCR showed *SESN3* expresion was evidently enhanced after SESN3 was delivered by a lentiviral vector to Jurkat cells (Fig. 5D). This action significantly inhibited the growth and CFC production in Jurkat cells (Fig. 5E, 5F), suppressed mTOR/S6K/protein synthesis signaling, while activated AMPK (Fig. 5G‒I). Conversely, opposite results were obtained in loss-of-function experiments (Supplementary Fig. S9A‒E). It was worth to note that SESN3 silencing significantly shortened the survival of leukemic mice in a xenograft model as well (34 vs. 39 days, *p* < 0.001) (Fig. 3O). Since SESN3 enhancement contributed to the inhibition of T-ALL cell growth mediated by autophagy inhibition, the effect of SESN3 overexpression on autophagy activity was also studied. The data showed that SESN3 led to the accumulation of both p62 and LC3II (Fig. 5G), which resembled the effect of ATG4B silencing in T-ALL cells. Interestingly, SESN3 only decreased protein expression of ATG4B but not ATG5 (Fig. 5G), while the transcript expression of *ATG4B* or *ATG5* was not affected by SESN3 overexpression (Fig. 5J). In line with this, SESN3 silencing increased autophagic flux and elevated ATG4B but not ATG5 protein expression, while the transcript expression of *ATG4B* or *ATG5* was not altered (Supplementary Fig. S9D, 9 F). As SESN3 is an mTOR inhibitor, the effects of rapamycin on T-ALL cells was also analyzed. The results showed that mTOR signaling was inhibited as expected, and the protein expression of ATG4B but not ATG5 was suppressed (Fig. 5K), meanwhile *ATG4B* transcript was not affect by the treatment (Fig. 5L), suggesting that SESN3 inhibited ATG4B protein expression in an mTOR dependent manner. Taken together, these results strongly indicated that SESN3 plays a novel tumor-suppressor role in T-ALL possibly through its inhibition of both mTOR signaling and autophagy activity.

### Atg4b depletion has mild effects on normal hematopoiesis and T cell development

**Fig.6:**
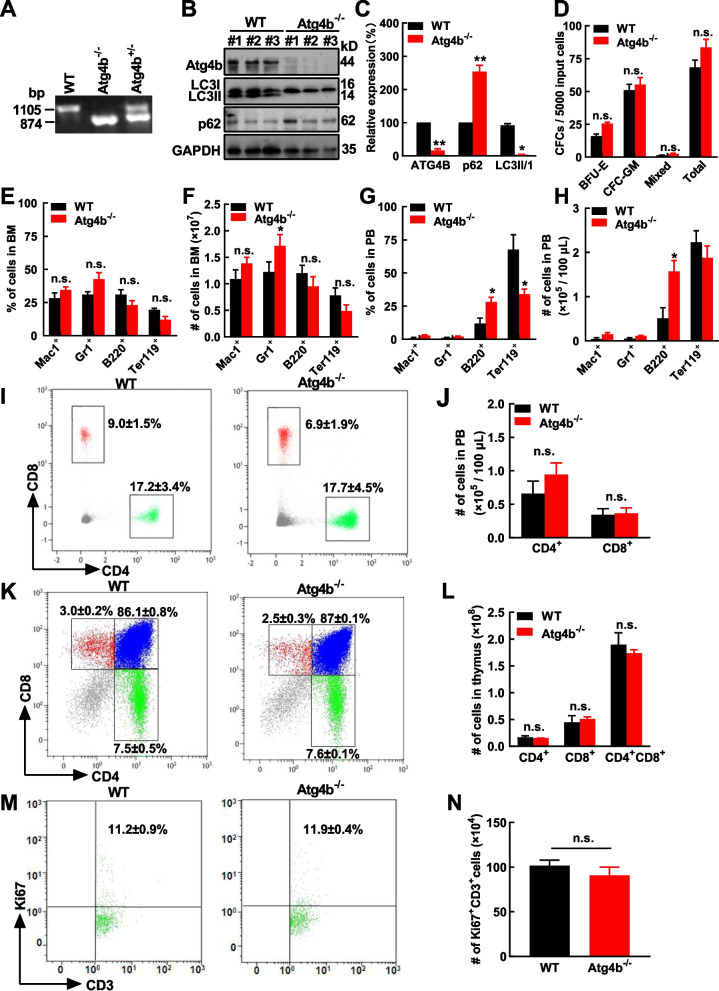
Atg4b deficiency has mild effects on normal hematopoietic cells. **A** Typical photo of the genotyping analyses of the wild-type (WT), *Atg4b*^±^, and *Atg4b*^−/−^mice is displayed. **B**,** C** The expression of Atg4b, p62, and LC3 in bone marrow (BM) cells from WT and *Atg4b*^−/−^mice was analyzed by Western blotting (**B**). and the expression of ATG4B, p62, and LC3II/I was analyzed statistically (**C**). **D** BM cells from WT and *Atg4b*^−/−^ mice were harvested, and then plated for colony-forming cell (CFC) assays (*n* = 3). **E**,** F** The percentages of Mac1^+^, Gr1^+^, B220^+^, and Ter119^+^ cells in the BM (from two femurs and two tibias) of WT and *Atg4b*^−/−^mice were compared (*n* = 5) (E), and the absolute numbers were analyzed as well (**F**). **G**,** H** The percentages of of Mac1^+^, Gr1^+^, B220^+^, and Ter119^+^ cells in the peripheral blood (PB) of WT and *Atg4b*^−/−^mice were compared (*n* = 5) (**G**), and the absolute numbers were counted as well (**H**). **I**,** J** The expression of CD4 and CD8 on the surface of peripheral blood (PB) cells from WT and *Atg4b*^−/−^ mice was analyzed by flow cytometry (I). The absolute numbers of CD4^+^ and CD8^+^ cells (per 100 μL PB) were quantified (*n* = 5) (**J**). **K**,** L** The expression of CD4 and CD8 on the surface of thymus cells from WT and *Atg4b*^−/−^ mice was analyzed by flow cytometry (**K**). The absolute numbers of CD4^+^, CD8^+^, and CD4^+^CD8^+^ cells were numerated (*n* = 5) (**L**). **M**,** N** CD3^+^ cells were enriched from WT and *Atg4b*^−/−^ mice, these cells were stimulated by CD3/28 and then cultured. 72 h later, Ki67 and CD3 staining was used to analyze cell proliferation (**M**), the absolute numbers of Ki67^+^CD3^+^ cells were counted (**N**). BFU-E, erythroid burst-forming unit; CFU-GM, granulocyte–macrophage colony-forming unit; Mixed, granulocyte-erythroid-macrophage-megakaryocyte, colony-forming unit. The data are presented as the means ± SEM. Student’s *t* test was used to estimate the statistical significance. *p < 0.05; n.s., not significant

To determine the effects of Atg4b loss on normal hematopoiesis and T cell development, Atg4b deficient (*Atg4b*^−/−^) mice were utilized in the present study. Genotyping and Western blotting analysis confirmed the loss of *Atg4b* allele and Atg4b expression (Fig. 6A‒C). Notably, slightly increased p62 was observed in the BM cells of *Atg4b*^−/−^ mice compared with WT mice (Fig. 6B, 6C), which indicated the impaired autophagy activity in Atg4b deficient cells. Meanwhile, Atg4b deficiency reduced LC3II, which suggested that the generation of LC3II in murine hematopoietic cells heavily depends on Atg4b activity. The differential effects of ATG4B depletion on LC3II/I level in murine and human cells possibly is due to the cell context-dependent balance between ATG4B’s activities of lipidation and delipidation. The major organs, including the heart, liver, and spleen, of both WT and *Atg4b*^−/−^ mice were analyzed (Supplementary Fig. S10A), and the coefficients (ratio of organ weight to body weight) of these organs in the two groups were not significantly different (Supplementary Fig. S10B). The CFC assay showed that the BM cells from the WT and *Atg4b*^−/−^ mice had similar proliferation and differentiation capacities (Fig. 6D).The BM cells of eight-week-old mice were analyzed using flow cytometry, the percentages of Mac1^+^, Gr1^+^, B220^+^, and Ter119^+^ cells were not altered (Fig. 6E), and the absolute numbers of them were not different between WT and *Atg4b*^−/−^ mice except for that of Gr1^+^ cells (Fig. 6F). Similar assays were performed with peripheral blood cells. The increased B220^+^ cells in terms of percentage and absolute number were observed, while decreased percentage of Ter119^+^ was detected (Fig. 6G, 6H). As the spleen is the largest secondary lymphoid organ in the body and plays an important role in the homeostasis of peripheral T cell [47], similar analysis was performed with the spleen and the results showed that Atg4b deficiency did not alter the percentages and absolute numbers of Mac1^+^, Gr1^+^, B220^+^, and Ter-119^+^ cells (Supplementary Fig. S11A, B).

Then the effect of Atg4b deficiency on T cell development was studied. Firstly, there was no evident difference in CD4 and CD8 subpopulations of T cells analyzed in both the PB and thymus between WT and *Atg4b*^−/−^ mice (Fig. 6I‒L). However, Atg4b deficiency reduced the presence of CD4^+^ and CD8^+^ cells in the spleen (Supplementary Fig. S11C, D). Therefore, the response of murine T cell to external stimulation was assessed, CD3^+^ cells from thymus were collected and then stimulated by CD3/28, the activation status (indicated by Ki67 expression) and the numbers of activated cells between WT and *Atg4b*^−/−^ cells were similar (Fig.6 M,6 N).

Lastly, our study showed that ATG4B silencing did not affect the growth of human CD3^+^ cells, and had mild effect on CFC production of human CD34^+^ cells (Supplementary Fig. S12). Overall, these data indicated that ATG4B depletion has mild effects on normal hematopoiesis and T cell development..

### ATG4B inhibitor treatment exhibits anti-leukemia activities in vitro and in vivo

**Fig.7:**
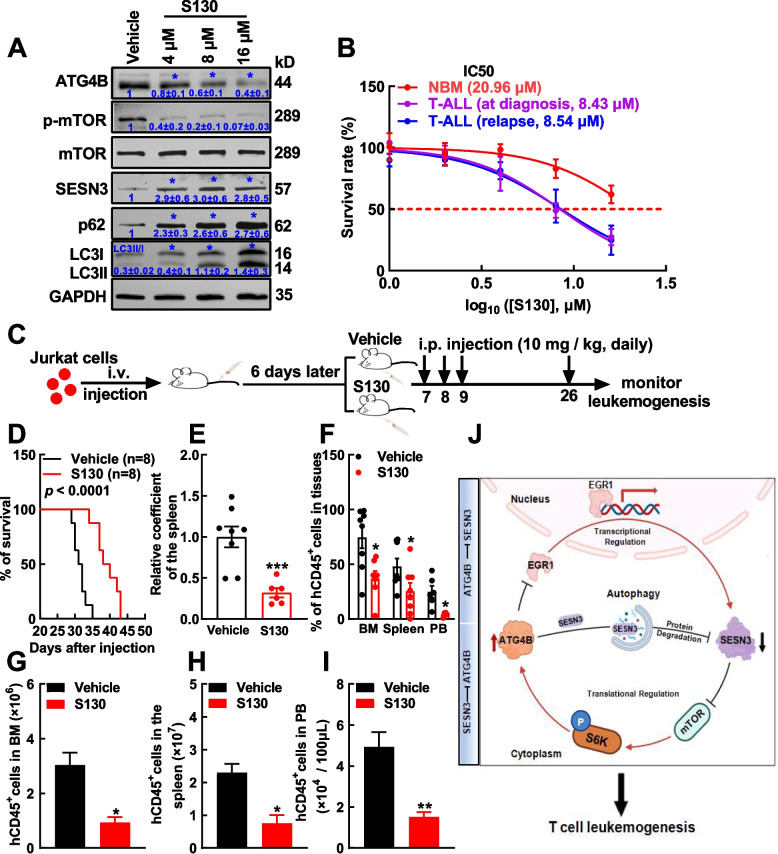
ATG4B inhibitor impairs T-cell leukemogenesis. **A** Jurkat cells were treated with S130, an ATG4B inhibitor, and then the cells were subjected to Western blotting to analyze the expression of ATG4B, p-mTOR, mTOR, SESN3, p62, and LC3. **B** Normal bone marrow (NBM) CD3^+^ cells from healthy donors (*n* = 4), BM cells from patients with T-ALL at diagnosis (*n* = 3) and in relapse (*n* = 3) were treated with various concentrations of S130 (0.5, 1, 2, 4, 8, and 16 μM), IC50 was estimated. **C** Schematic illustration of a human T-ALL xenograft is displayed. Jurkat cells were injected into immunodeficient mice through the tail vein (8 × 10^6^ cells/mouse and eight mice in each group). Six days after engraftment, S130 (10 mg/kg) or normal saline was administrated to each group for 20 consecutive days. **D** The Kaplan‒Meier method was used to study the survival of these groups of mice, and the *p* value was estimated using the log-rank test. **E** The relative coefficients (ratios of spleen weight to body weight) in S130-treated group were compared with those in the control (vehicle) group. **F** Leukemic cells (hCD45^+^) in the BM, spleen, and peripheral blood (PB) were analyzed by flow cytometry and summarized statistically. **G**–**I** The absolute numbers of leukemic cells in the BM (**G**), spleen (**H**), and PB (**I**) from these groups were compared. **J** The cartoon deciphers the circuit of ATG4B and SESN3 in T-ALL cell. The highly expressed ATG4B increases autophagy in T-ALL cells and leads to autophagy degradation of SESN3. Additionally, ATG4B impairs EGR1-mediated transcriptional activation of SESN3. These actions result in the decreased SESN3, which enhances mTOR/S6K-mediated protein synthesis, including ATG4B itself to promote T-cell leukemogenesis. The data are presented as the means ± SEM, and the statistical significance of the differences was estimated with Student’s *t* test. *p < 0.05; **p < 0.01; ***p < 0.001

In the present study, S130 was evaluated for its anti-leukemia activity. First, S130 treatment in Jurkat cells led to LC3II accumulation and p62 enhancement in a dose-dependent manner (Fig. 7A), which was reminiscent of previous data of ATG4B silencing. Meanwhile, SESN3 expression was increased and mTOR signaling was inhibited in a dose-dependent manner (Fig. 7A). Importantly, S130 exhibited stronger inhibitory effects on the viability of leukemic cells from patients with T-ALL at diagnosis or in relapse than on that of normal CD3^+^ cells (Fig. 7B).

Jurkat cells were used to evaluate the anti-leukemia activity of S130 in a xenograft model. Following the experimental procedure design (Fig. 7C), the results showed that S130 significantly prolonged the survival of leukemic mice in comparison to control (vehicle) mice (40 vs. 32 days, *p* < 0.0001, Fig. 7D). After the diseased mice were dissected, the coefficient of the spleen in the S130-treated group was significantly lower than that in the control group (Fig. 7E). S130 treatment significantly decreased the infiltration of leukemic cells in the BM, spleen, and PB, as determined by flow cytometry (Fig. 7F‒I, Supplementary Fig. S13).

Moreover, the efficacy of S130 was evaluated in a PDX model by IV injection of human T-ALL patient cells. Interestingly, S130 significantly prolonged the survival of leukemic mice (38 vs. 35 days, *p* < 0.01), decreased the coefficient of the spleen, and reduced leukemic cell infiltration in the BM, spleen, and PB (Supplementary Fig. S14). This PDX model held to a lesser extent when comparing with the Jurkat cell-generated xenograft model.

To summarize the present study, a working model is proposed. The increased autophagy activity induced by highly expressed autophagy-related genes including *ATG4B* decreases the expression of SESN3, a novel tumor-suppressor in T-ALL through autophagy-mediated protein degradation and EGR1-regulated transcription. The decreased SESN3 enhances mTOR/S6K/protein synthesis, likely including ATG4B itself. Therefore, the circuit composed of ATG4B and SESN3 promotes T cell leukemogenesis (Fig. 7J). Our study also demonstrates that targeting ATG4B leads to SESN3 enhancement to inhibit both mTOR/S6K signaling and ATG4B-mediated autophagy, which hampers T-ALL progression and possibly presents a new option for the disease treatment.

## Discussion

In the present study, the increased MDC staining of T-ALL cells and enhanced expression of *ATG4s* and *ATG5* in these cells strongly suggested that autophagy activity promoted T-ALL cell growth. Among all these tested genes, *ATG4B* has the highest relative expression; additionally, *Atg5*^−/−^ mice die immediately after birth [15, 48] while *Atg4b*^−/−^ mice are viable [25]. Therefore, we focused on ATG4B in this study. The role of ATG4B in cancer cells is well documented [23, 26–28]; however, studies on ATG4B in hematological malignancies are limited [16]. To the best of our knowledge, our study provides the first piece of evidence that ATG4B is aberrantly expressed in T-ALL cells and sustains the growth of these cells. Given that the treatment progress of T-ALL is relatively slow, the evidence that ATG4B inhibitors impair T-ALL cell growth might promote targeting ATG4B as a new option for T-ALL treatment.

Although Atg4b knockout mice are viable and behave normally in several tests [25], suggesting Atg4b as an appropriate therapeutic target; however, the effects of ATG4B on normal hematopoiesis and T cell development remain unclear. Our data showed that Atg4b deficiency had subtle effects on hematopoietic cells. Atg4b defects had opposite effects on B220^+^ and Ter119^+^ cells in the PB, which requires further investigation. Atg4b defects did not affect T cells in the peripheral blood and thymus or their response towards CD3/28 stimilation. Moreover, RNA interference-mediated gene silencing showed that ATG4B inhibition did not affect CFC production in human CD34^+^ cells or the growth of human CD3^+^ cells from healthy individuals. Nevertheless, Atg4b defects reduced T cells in the spleen, which was possibly caused by perturbation of the migration/homing ability of T cells or the abnormal secretion of cytokines from myeloid cells. The clear mechanism needs more studies to clarify. Overall, targeting ATG4B has mild effects on hematopoietic cells, including T cells, which supports the utility of ATG4B as a suitable therapeutic target for hematological malignancies.

Although several studies have demonstrated the critical role of ATG4B in tumor cells and the utility of ATG4B inhibitors in tumor inhibition [23, 26–28], the underlying mechanism by which ATG4B blockade hampers tumor cell growth has not been fully studied. In the present study, RNA-seq data were generated. Multiple molecular pathways were perturbed upon this action, including the Wnt/catenin, NF-κB, and MYC pathways, whose critical roles in T-ALL have been reported [49–52]. Some cellular pathways, including protein translation, were also perturbed. Subsequent validation led to the investigation of SESN3, which was significantly up-regulated upon ATG4B blockade. Mechanistically, our data showed that SESN3 expression was regulated by autophagy activity in T-ALL cells, starvation-induced autophagy immediately decreased SESN3 protein expression without altering its transcript expression. Conversely, inhibition of autophagy by S130, an ATG4B inhibitor in a short period of time increased SESN3 protein expression without deviating its transcript expression. However, when the time of S130 exposure was long enough, both protein and transcript expression of SESN3 was increased, which resembled the scenario in a typical lentiviral transduction experiment. Moreover, EGR1 was identified to play a critical role in SESN3 transcriptional activation upon ATG4B blockade. It is worth to note that EGR1-mediated SESN3 transcriptional activation has not been reported.

Our study demonstrate that SESN3 is significantly down-regulated in T-ALL patient samples and acts as a novel tumor-suppressor in T-ALL, which is in line with the fact that SESN3 suppresses the growth of BCR-ABL^+^leukemic cells [35]. Since SESN3 inhibits mTORC1, it was plausible to observe that SESN3 overexpression inactivated mTOR/S6K/protein synthesis pathway while activated AMPK. Given that mTORC1 activation is a hallmark of T-ALL [53–56], the inactivation of mTORC1 contributes to the tumor-suppressor role of SESN3 in T-ALL cells. Nevertheless, the rescue experiment was performed to show that SESN3 silencing partially reversed ATG4B silencing-induced growth inhibition in vitro and in vivo (Fig. 3N, 3O), which implied that SESN3 silencing possibly increased autophagy activity. Therefore, the effect of SESN3 on autophagy activity was studied and the results showed that SESN3 inhibited autophagy activity, which was not compatible with the concept that mTOR inhibition leads to autophagy activation through the regulation of ULK1 [57]. To clarify this discrepancy, rapamycin was used to treat T-ALL cells and the data showed that both SESN3 overexpression and rapamycin treatment resulted in the decreased ATG4B expression at posttranscriptional level but not ATG5. Functionally, SESN3 silencing-induced growth promotion was reversed by ATG4B silencing or S130 treatment (Fig. 3N, 3O; Supplementary Fig. S7A, 7B). These results allowed us to speculate that SESN3 or rapamycin inhibited mTOR and activated autophagy initiation, however decreased S6K signaling specifically inhibited ATG4B translation, and these two effects ended up with autophagy flux decrease. Considering SESN3 expression was regulated by autophagy activity, our work revealed a novel signaling circuit composed of ATG4B and SESN3 to promote T cell leukemogenesis.

In conclusion, our data demonstrate that autophagy plays a critical role in T-ALL progression and suggest that targeting ATG4B is a new option for T-ALL treatment.

## Supplementary Information

Supplementary Material 1.

## Data Availability

Data supporting the findings are included in this article and the Supporting Information. RNA-seq data have been deposited in Gene Expression Omnibus and assigned the accession number as GSE268628.
