# Supplementary material for: A novel regulatory circuit of ATG4B and SESN3 promotes T cell leukemogenesis
Source: J Exp Clin Cancer Res. 2025 Nov 22;44:322. doi: 10.1186/s13046-025-03588-0 (PMC12723951; doi:10.1186/s13046-025-03588-0)
Supplement: Supplementary file 1 — Supplementary Material 1. [file 13046_2025_3588_MOESM1_ESM.pdf]

Supporting information

A novel regulatory circuit of ATG4B and SESN3 promotes T cell leukemogenesis

Wenjuan Ma,[a,*] Lei Zhang,[a,*] Haixia Zhou,[b,*] Xiuyan Zhang,[a,*], Xingjie Qin,[a] Yan Wan,[a] Rongyao Ma,[a] Xueyan Song,[a] Xiaonan Zhou,[a] Hong Liu,[b] Bo Hu,[c] Depei Wu,[b,c] Jianrong Wang,[a,d] Xiaoyan Jiang,[e] Yun Zhao[a,d,f]

[a]Cyrus Tang Medical Institute, National Clinical Research Center for Hematologic Diseases, Collaborative Innovation Center of Hematology, Soochow University, Suzhou 215123, China

[b]The First Affiliated Hospital of Soochow University, Jiangsu Institute of Hematology, Suzhou 215006, China

[c]Institute of Blood and Marrow Transplantation, Soochow University, Suzhou, China

[d]Suzhou Ninth Hospital Affiliated to Soochow University, Suzhou 215200, China

[e]Terry Fox Laboratory, British Columbia Cancer Research Institute and Department of Medical Genetics, University of British Columbia, Vancouver, BC, V5Z 1L3, Canada

[f]NHC Key Laboratory of Thrombosis and Hemostasis, MOE Engineering Center of Hematological Disease, Soochow University, Suzhou 215006, China

* These authors contributed equally to this work.

Supplementary Table S1 The characteristics of T cell acute lymphoblastic leukemia patients recruited in this study.

		**Patients**
No.	***Total***	**53**
	F	**39**
	M	**14**
Age	***Mean***	**33.4**
	Range	**11~80**
	Age＜50, n(%)	**40(75.5)**
	Age≥50, n(%)	**13(24.5)**
WBC, ×10^9/L	***Mean***	**76.7**
	Range	**2.87~369**
	Missing, n(%)	**10(18.8)**
Hb, g/L	***Mean***	**89.8**
	Range	**39~141**
	Missing, n(%)	**10(18.8)**
Plt, ×10^9/L	***Mean***	**87.6**
	Range	**5~311**
	Missing, n(%)	**10(18.8)**

WBC, white blood cell; Hb, hemoglobin; Plt, platelet.

Supplementary Table S2 The primers used in this study for RT–qPCR analysis.

**Genes**	**Sequence of primers**	**Amplicon sizes (bp)**
β-ACTIN	CACCATTGGCAATGAGCGGTTCC	90
	GTAGTTTCGTGGATGCCACAGG	
ATG4A	CCAAGCCAGAAGTGACAACCA	109
	GACAGACCTTCAAGTTGAGTTC	
ATG4B	ACTGGGAAGATGGACGCAGC	94
	AGTATCCAAACGGGCTCTGAG	
ATG4C	TGTTCAGGACTTCAAACGAGCT	81

	TCTCTGGAATGACCATTTACAAAA	
ATG4D	GTGGTGTACGTTTCTCAGGAC	104
	CACCAGGATGACCACAGACTT	
ATG5	CAGATGGACAGTTGCACACAC	127
	AGGTGTTTCCAACATTGGCTCA	
ATG7	TTGCCCACAGCATCATCTTCG	124
	ACTGAGGTTCACCATCCTTGG	
BECLIN-1	GGAGAGGAGCCATTTATTGAAAG	103
	AGAGTGAAGCTGTTGGCACTTT	
ATG12	GGAAGGACTTACGGATGTCTC	141
	AGGAGTGTCTCCCACAGCC	
MAP1LC3B	GAACGATACAAGGGTGAGAAGC	132
	AGAAGGCCTGATTAGCATTGAG	
SESN3	GACAGTGACCTGCTATCCTGAG	154
	CCGAGTTATGGCACGAAGAGCA	
HES4	GAGCGCGTATTAACGAGAGCCT	118
	CTCACGGTCATCTCCAGGATGT	
METTL5	GCCCAAGCTACTTCTGGAACAG	141
	CCGATGCTAAGTACTCCACAACC	
DSCC1	CGCTGAGTTTCAAGAAGTGTGGC	143
	CCTCAGGTAAATCATCTACTTTCAG	
CCPG1	CAGAAGCGTCAACAGTTAGTCAG	156
	CTCTGCTTCAGTAAGTGTCCAAC	
β-actin	GAGACCTTCAACACCCCAGCCA	92
(mouse)	GAGTCCATCACAATGCCTGTG	
Atg4b	TGATACTCTCCGGTTTGCTGA	123
(mouse)	CTGGATGCAACATCAGACAAG	

Supplementary Table S3 The antibodies used for Western blotting

Antibodies	Information
anti-ATG4B	ab70867, Abcam, Cambridge, MA, USA
anti-p62	PM045, MBL, Japan
anti-LC3	NB1002220, N0VUS, Colorado ,USA
anti-SESN3	A5164, ABclonal
anti-S6K	A2190, ABclonal
anti-p-S6K	AP0564, ABclonal
anti-GAPDH	60004-1-Ig, Proteintech, Rosemont, IL, USA
anti-AMPK	A12718, ABclonal
anti-p-AMPK	2535S, CST, Boston, USA
anti-puromycin	A21205, ABclonal
anti-ATG5	A19677, ABclonal
anti-p-mTOR	67778-1-Ig, Proteintech, Rosemont, IL, USA
anti- mTOR	6688-1-Ig, Proteintech, Rosemont, IL, USA

Supplementary Table S4 The shRNA sequences used in this study.

Genes	Sequences of shRNAs
ATG4B	GAAGCTTGCTGTCTTCGATAC (#1)
	GAAGCTTGCTGTCTTCGATAC (#2)
SESN3	GGCACTATATTGCAATAATGG (#1)
	GCTGCGCAGAGCTTTATTTAA (#2)

Supplementary Table S5 The sgRNA sequences used in this study.

Genes	Sequences of sgRNAs
ATG4B	TCCTGTCGATGAATGCGTTGAGG (#1)
	AGCAAACCGGAGAGTGTCGTAGG (#2)
NC	GAAGATGGGCGGGAGTCTTC

Supplementary Table S6 The primer sequences used for the genotyping in this

study.

Primers	Sequences
Atg4b-F	AAGATGGAAGGGTGACCCTTCCT
Atg4b-R	CCTATCCAGCACAGAGCCTATCTC

Supplementary Table S7 The differentially expressed transcripts upon ATG4B silencing in Jurkat cells identified by RNA-seq.

Gene ID	Gene Symbol	log2 (shATG4B / Scrambled)	Qvalue (shATG4B / Scrambled)
51015	*ISOC1*	-2.125982217	1.02E-16
23192	*ATG4B*	-1.808910981	5.96E-14
10966	*RAB40B*	-1.341455932	4.85E-04
81557	*MAGED4B*	-1.300170019	0.034114788
29081	*METTL5*	-1.298818421	1.29E-04
90362	*FAM110B*	-1.296090923	0.015147555
10440	*TIMM17A*	-1.131815463	2.13E-05
653489	*RGPD3*	-1.079079785	1.92E-05
4052	*LTBP1*	-1.067910402	0.002604039
10296	*MAEA*	-1.057185224	2.61E-05
7292	*TNFSF4*	-1.03727174	4.08E-05
57801	*HES4*	-1.034984924	0.035238551
11275	*KLHL2*	-1.031884158	3.62E-05
79075	*DSCC1*	-1.018580701	1.15E-04
10523	*CHERP*	-1.002481254	9.15E-12
79094	*CHAC1*	-0.996301221	0.032272994
112812	*FDX2*	-0.940892588	0.04131118
25979	*DHRS7B*	-0.937333755	0.024594518
133686	*NADK2*	-0.894358221	1.10E-06
3638	*INSIG1*	-0.891825914	0.017120346
80224	*NUBPL*	-0.880373027	4.99E-05
58488	*PCTP*	-0.875599865	0.031800859
132158	*GLYCTK*	-0.86023027	0.039647631
474343	*SPIN2B*	-0.831376644	0.043542188
29796	*UQCR10*	-0.827127434	0.043133065
11261	*CHP1*	-0.804465005	1.34E-08
28969	*BZW2*	-0.804097682	8.24E-04
55726	*INTS13*	-0.776445082	0.004688582
51202	*DDX47*	-0.747173356	0.028957945
375035	*SFT2D2*	-0.73871106	8.46E-04
29916	*SNX11*	-0.724263805	0.023981867

10007	*GNPDA1*	-0.698238394	1.29E-04
55135	*WRAP53*	-0.683552892	0.043133065
2280	*FKBP1A*	-0.67875136	0.018502233
55884	*WSB2*	-0.677727419	0.008894913
4287	*ATXN3*	-0.671212491	0.004426565
5062	*PAK2*	-0.645516371	6.27E-04
399664	*MEX3D*	-0.640932256	0.034114788
8934	*RAB29*	-0.636672257	0.003888809
1845	*DUSP3*	-0.608245169	0.014726615
7913	*DEK*	-0.607000512	0.014726615
9054	*NFS1*	-0.558824245	0.045444741
55156	*ARMC1*	-0.557303469	0.033745599
79109	*MAPKAP1*	-0.547656575	0.006566661
58480	*RHOU*	-0.547511739	0.008894913
2534	*FYN*	-0.539913799	6.58E-04
26469	*PTPN18*	-0.532734837	0.015646937
149986	*LSM14B*	-0.517735796	0.015991921
10055	*SAE1*	-0.507627244	0.043526881
8565	*YARS1*	-0.481616986	0.019186596
113251	*LARP4*	0.41024343	0.034050257
79718	*TBL1XR1*	0.430451148	0.015485213
114789	*SLC25A25*	0.497600936	0.049087926
51271	*UBAP1*	0.503859674	0.037218426
7415	*VCP*	0.507157023	0.006914762
84804	*MFSD9*	0.513026431	0.046334274
5214	*PFKP*	0.521027525	0.023944276
79183	*TTPAL*	0.521591974	0.037218426
51429	*SNX9*	0.527092683	0.016651161
5717	*PSMD11*	0.528654388	0.004661474
200014	*CC2D1B*	0.528875434	0.016434038
3423	*IDS*	0.547714368	0.007278157
51164	*DCTN4*	0.547755591	0.014215212
5707	*PSMD1*	0.55376353	0.001648832
152006	*RNF38*	0.554406132	0.012561856
5708	*PSMD2*	0.555342962	0.001648832
23022	*PALLD*	0.556072319	0.006914762
29959	*NRBP1*	0.556872518	0.009666973
8323	*FZD6*	0.569312208	0.030195589
8408	*ULK1*	0.585667528	0.01701969
80155	*NAA15*	0.588652878	0.039566356
23032	*USP33*	0.592069785	0.00498535
29110	*TBK1*	0.592895598	0.007734058
5660	*PSAP*	0.593915827	0.049087926

3964	*LGALS8*	0.594286758	0.02998354
26580	*BSCL2*	0.597907203	0.044575679
4097	*MAFG*	0.602842577	0.009666973
23198	*PSME4*	0.615916294	0.048825816
26750	*RPS6KC1*	0.617294784	0.004456093
54545	*MTMR12*	0.619558923	1.29E-04
23731	*TMEM245*	0.621259826	0.029257588
37	*ACADVL*	0.623194253	0.001104803
5130	*PCYT1A*	0.629582687	0.003240573
22862	*FNDC3A*	0.633407519	0.008894913
55666	*NPLOC4*	0.63929348	6.06E-04
3984	*LIMK1*	0.648356234	0.002129444
30001	*ERO1A*	0.65126867	9.70E-05
55432	*YOD1*	0.651710119	0.006633429
2678	*GGT1*	0.659151992	0.009244948
79735	*TBC1D17*	0.66393211	0.002910244
84056	*KATNAL1*	0.676675819	0.043526881
7525	*YES1*	0.693440911	0.002001996
55652	*SLC48A1*	0.703180611	0.009699358
10962	*MLLT11*	0.703362668	0.009666973
54480	*CHPF2*	0.705383417	0.002129444
5252	*PHF1*	0.706115586	8.74E-04
535	*ATP6V0A1*	0.71100767	0.023981867
64506	*CPEB1*	0.713887025	0.001736709
8754	*ADAM9*	0.714917369	0.001677256
51706	*CYB5R1*	0.721746056	0.022236424
4674	*NAP1L2*	0.72700204	0.019186596
4591	*TRIM37*	0.727806025	1.06E-04
2764	*GMFB*	0.728586255	2.31E-05
9815	*GIT2*	0.730956243	0.009243912
9055	*PRC1*	0.734891062	2.13E-05
767	*CA8*	0.737577932	2.13E-05
84803	*GPAT3*	0.740994848	0.01978366
140809	*SRXN1*	0.743107919	0.001289013
7171	*TPM4*	0.744400105	1.74E-06
64778	*FNDC3B*	0.747805357	0.039016242
283209	*PGM2L1*	0.755653081	0.00692448
94097	*SFXN5*	0.759535123	0.012079838
9253	*NUMBL*	0.771910922	0.027718803
284716	*RIMKLA*	0.778266986	0.002977214
5756	*TWF1*	0.778691642	0.015668912
4735	*SEPTIN2*	0.782907697	2.28E-06
6256	*RXRA*	0.785007694	0.021133056

23780	*APOL2*	0.78967701	1.79E-05
943	*TNFRSF8*	0.790066225	0.046665835
56262	*LRRC8A*	0.796846013	0.012425907
28964	*GIT1*	0.811398057	6.24E-04
23405	*DICER1*	0.84242913	0.039016242
1509	*CTSD*	0.845758495	2.13E-05
6041	*RNASEL*	0.851355088	0.001496904
402	*ARL2*	0.872081424	0.013220892
5597	*MAPK6*	0.895578423	0.001687767
56675	*NRIP3*	0.903627397	0.008894913
4644	*MYO5A*	0.945209243	0.02998354
64718	*UNKL*	0.952412972	3.70E-10
57659	*ZBTB4*	0.954183784	0.004322589
302	*ANXA2*	0.963578041	0.01116487
22924	*MAPRE3*	0.998426388	2.88E-04
2583	*B4GALNT1*	1.003838086	0.04583396
10058	*ABCB6*	1.049179552	9.67E-07
2017	*CTTN*	1.142862442	1.49E-05
2635	*GBP3*	1.217162695	4.61E-12
203259	*FAM219A*	1.228612153	4.50E-09
1958	*EGR1*	1.261962466	0.019186596
7277	*TUBA4A*	1.299193885	2.05E-05
968	*CD68*	1.342398145	0.007615074
143686	*SESN3*	1.343192198	1.29E-04
23710	*GABARAPL1*	1.397564257	6.01E-22
8542	*APOL1*	1.460249211	6.56E-08
1780	*DYNC1I1*	1.460946854	2.88E-04
10561	*IFI44*	1.497725976	1.33E-05
9856	*KIAA0319*	1.537135981	7.98E-10
4359	*MPZ*	1.581856918	1.33E-05
3601	*IL15RA*	1.89051367	0.018519229
29948	*OSGIN1*	1.909799356	1.33E-05
400629	*TEX19*	2.025223367	2.90E-21
654346	*LGALS9C*	2.025621229	0.004549729
8973	*CHRNA6*	2.464464086	0.016651161
55106	*SLFN12*	2.649157004	0.04180485
10333	*TLR6*	2.694212415	0.003407291
1191	*CLU*	2.746486444	6.71E-10
1608	*DGKG*	3.360298152	2.56E-07
100526767	*RNF103-CHMP3*	6.142224273	0.00402757

Supplementary Table S8 The Pathways Regulated by ATG4B through GSEA Analyses

**Enrichment plot**	**NES**	***p* value**	**FDR**
cell migration	1.72	< 0.01	0.16
mitotic cell cycle	1.67	0.007	0.19
Phagosome	1.84	<0.01	0.09
Lysosome	1.83	<0.01	0.04
canonical Wnt signaling pathway	1.84	<0.01	0.13
NIK/NF-kappa Bsignaling	1.84	0.001	0.14
ERK1 and ERK2 cascade	1.75	0.005	0.005
Ras protein signal transduction	1.63	0.007	0.22
Genes in the cancer module 27	1.58	<0.01	0.15
Cell cycle genes with peak expression in G1/S check point	-1.75	<0.01	0.05
Protein biosynthesis and ribosomes	-2.34	<0.01	0
Ribosome	-2.35	<0.01	0
cytoplasmic translation	-2.90	<0.01	0
Eukaryotic Translation Initiation	-2.91	<0.01	0
Genes involved in 3'-UTR -mediated translational regulation	-2.91	<0.01	0
Peptide chain elongation	-2.84	<0.01	0
MYC	-1.66	<0.01	0.08

GSEA, Gene Set Enrichment Analysis

Fig. S1

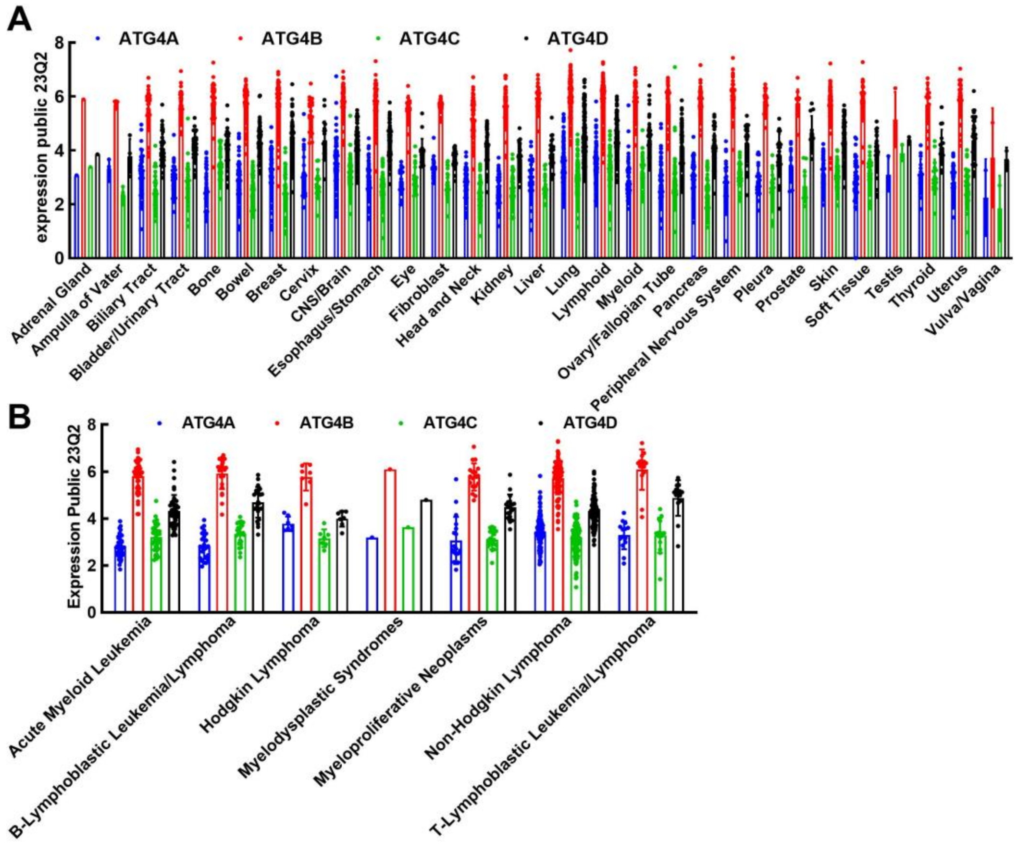

Supplementary Fig. S1 The gene expression of *ATG4* family was analyzed among 1036 human cancer cell lines in CCLE database.

Fig. S2

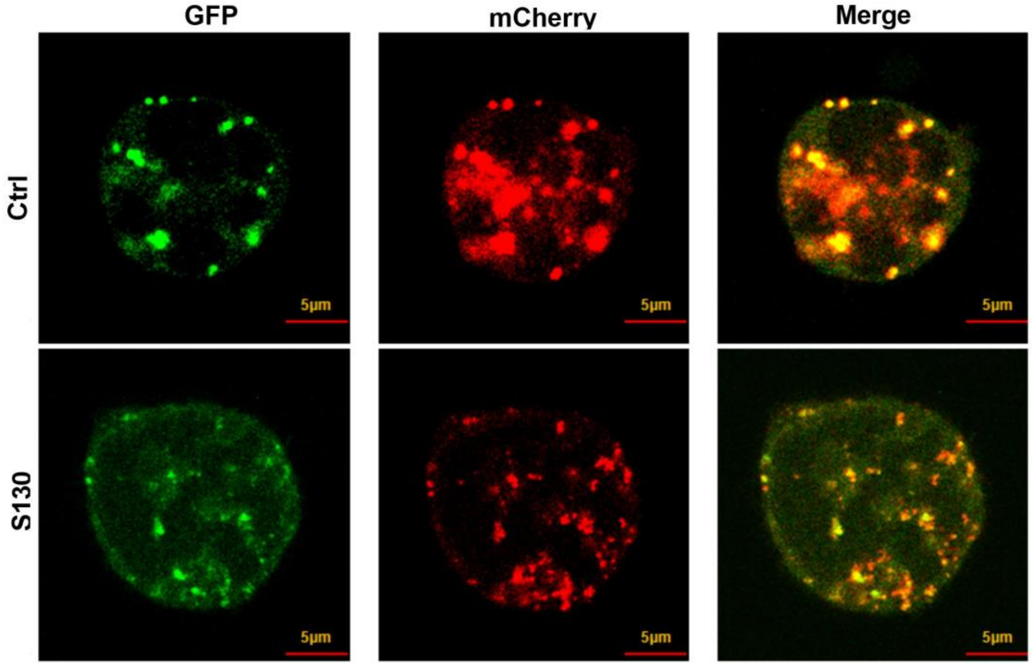

Supplementary Fig. S2 Confocal analysis of Jurkat cells using mCherry-GFP-LC3B vector. Jurkat cells were transduced by a retroviral vector containing mCherry-GFP-LC3B, then S130-treated and control cells were analyzed by confocal microscopy. Representative images are shown. Scale bar = 5 μm

Fig. S3

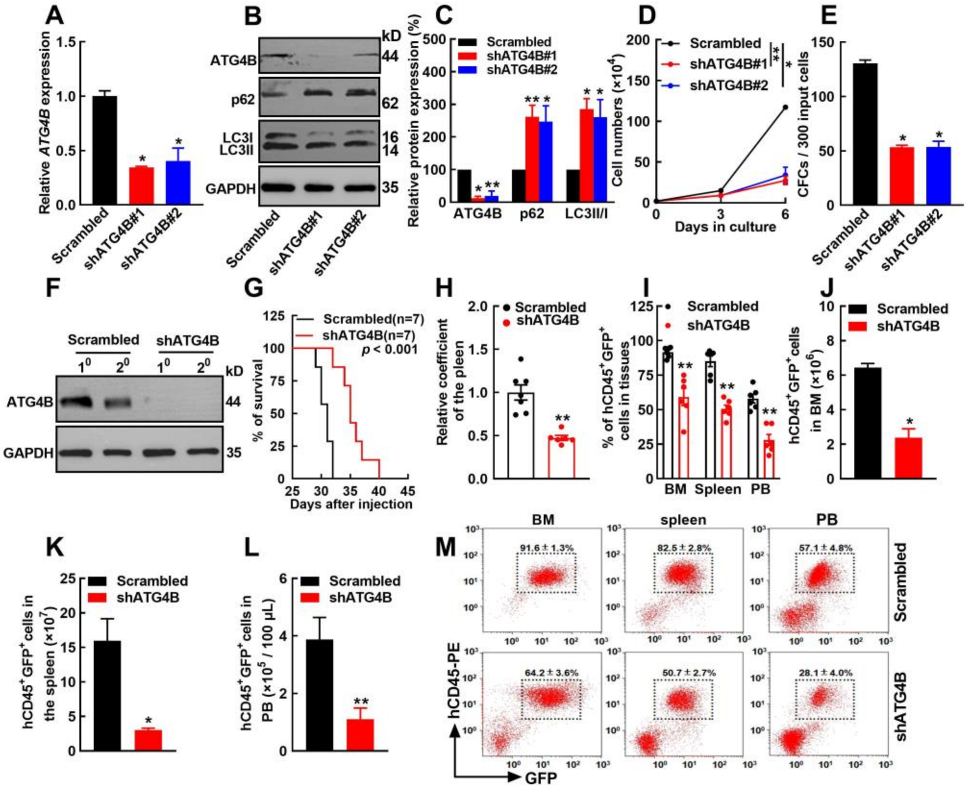

Supplementary Fig. S3 The effects of ATG4B silencing on MOLT-4 cells. (**A**–**E**) MOLT-4 cells were transduced with control (Scrambled), shATG4B#1, and shATG4B#2 lentiviruses, and these cells were subjected to RT–qPCR (n = 4, A), Western blotting (B, C), growth (n = 4, D), and colony-forming cell (CFC) (n = 4, E) analyses. (**F, G**) ATG4B-silenced and control MOLT-4 cells were injected into immunodeficient mice through the tail vein (7×10^6 cells/mouse and seven mice in each group). Leukemic cells ($hCD45^+GFP^+$) from two individual mice in control and shATG4B groups were analyzed for ATG4B expression by Western blotting (F). The Kaplan–Meier method was used to assess the survival, and the *p* value was estimated using the log-rank test (G). (**H**) The relative coefficients of the spleen (ratios of spleen weight to body weight) in control and shATG4B groups were compared. (**I**) The infiltration of leukemic cells in the bone marrow (BM), spleen, and peripheral blood (PB) of these two groups of mice were analyzed by flow cytometry and summarized statistically. (**J**–**L**) The absolute numbers of leukemic cells in the BM (J), spleen (K),

and PB (L) from ATG4B-silenced and control groups are displayed. (**M**) Representative graphs of flow cytometry to analyze the infiltration of leukemic cells in the BM, spleen, and PB from ATG4B-silenced and control groups of mice are shown. The data are presented as the means ± SEM. Student's *t* test was used to estimate the statistical significance. *$p < 0.05$; **$p < 0.01$.

Fig. S4

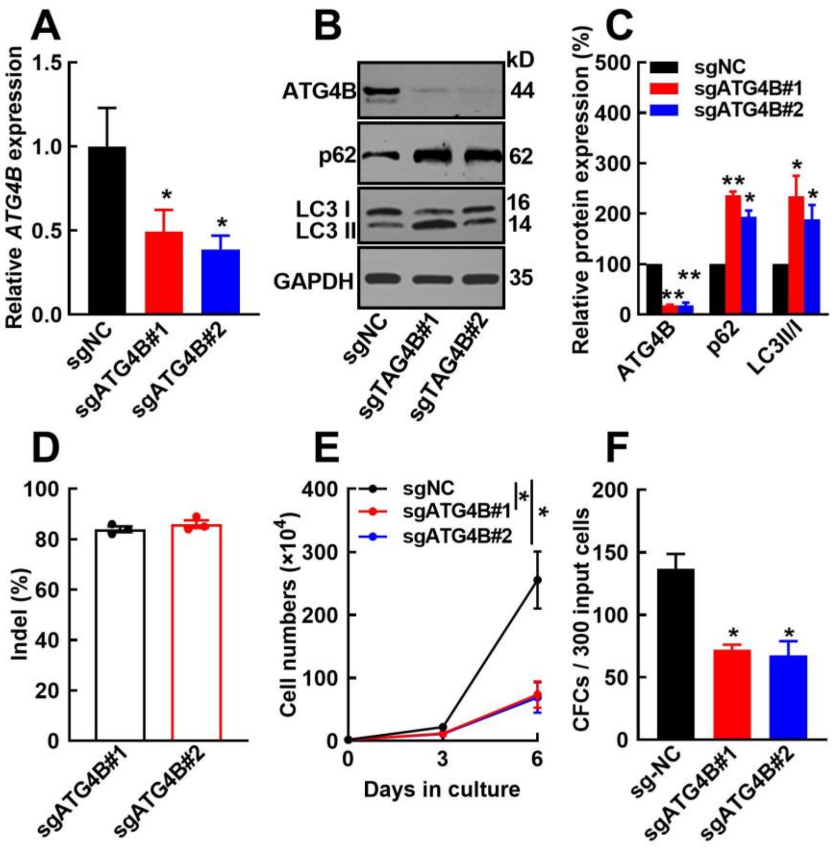

Supplementary Fig. S4 sgATG4B inhibits the growth of Jurkat cells. (**A**–**C**) sgRNA sequences against ATG4B (sgATG4B#1 and sgATG4B#2) or the control (sgNC) were delivered to Cas9 expressed Jurkat cells. Then these cells were subjected to RT–qPCR (n = 4, A) and Western blotting (B) analyses. The quantification of Western blotting is shown (C). (**D**) The insertion or deletion (Indel) of these cells were analyzed by DNA sequencing and summarized (n = 3). (**E, F**) The effects of sgATG4B on the growth (n = 4, E) and colony-forming cell (CFC) production (n = 4, F) were analyzed as well. The data are presented as the means ± SEM. Student's *t* test was used to estimate the statistical significance. *$p < 0.05$, **$p < 0.01$.

Fig. S5

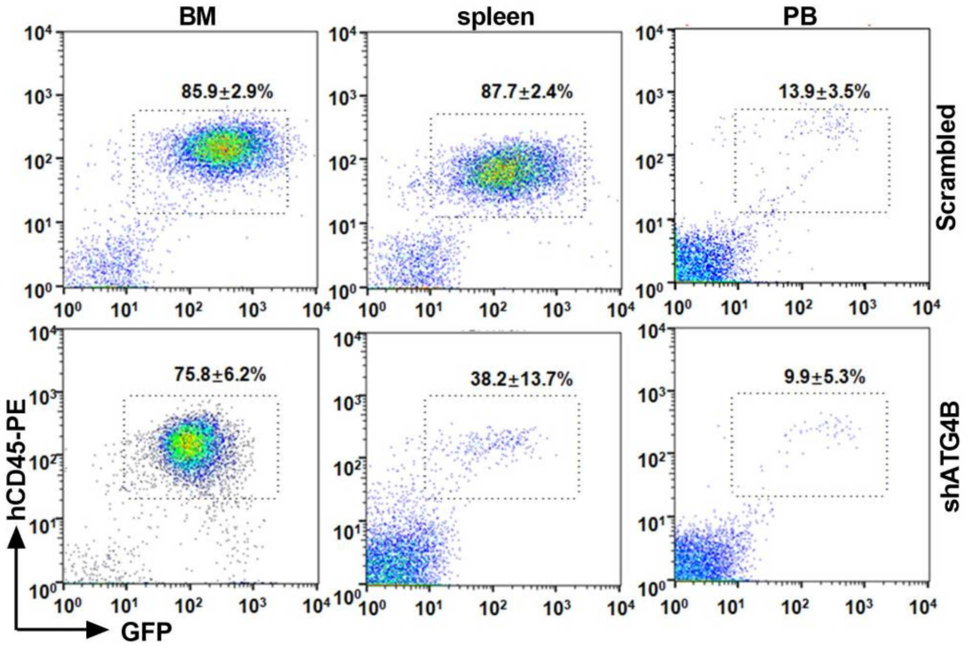

Supplementary Fig. S5 Flow cytometry analysis of the infiltration of leukemic cells. In this experiment, Jurkat cells were used to generate cell line-derived xenograft model. The infiltration of leukemic cells in the bone marrow (BM), spleen, and peripheral blood (PB) of ATG4B-silenced (shATG4B) and control (Scrambled) groups of mice were analyzed by flow cytometry, representative graphs are shown. The data are presented as the means ± SEM.

Fig. S6

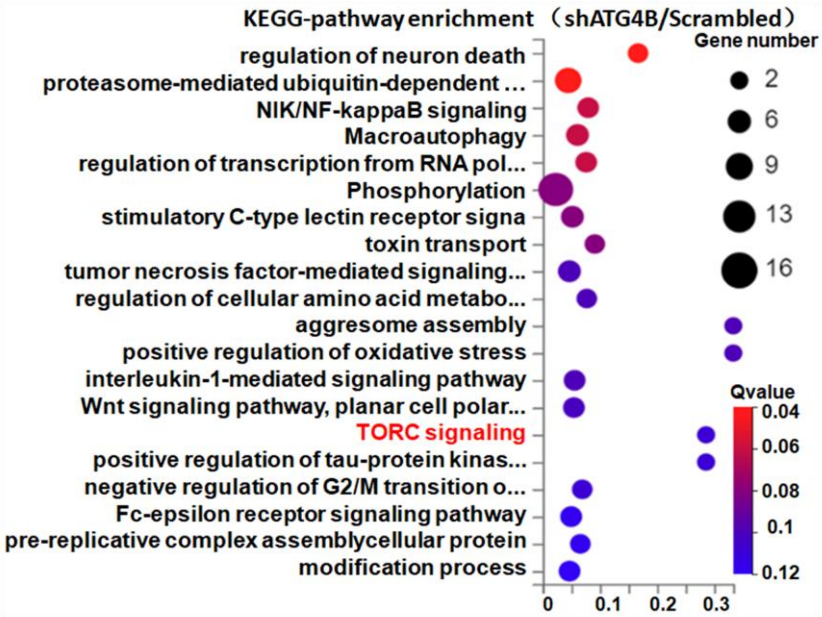

Supplementary Fig. S6 Kyoto Encyclopedia of Genes and Genomes (KEGG) pathway enrichment analysis of differentially expressed transcripts comparing ATG4B-silenced Jurkat cells with their control.

Fig. S7

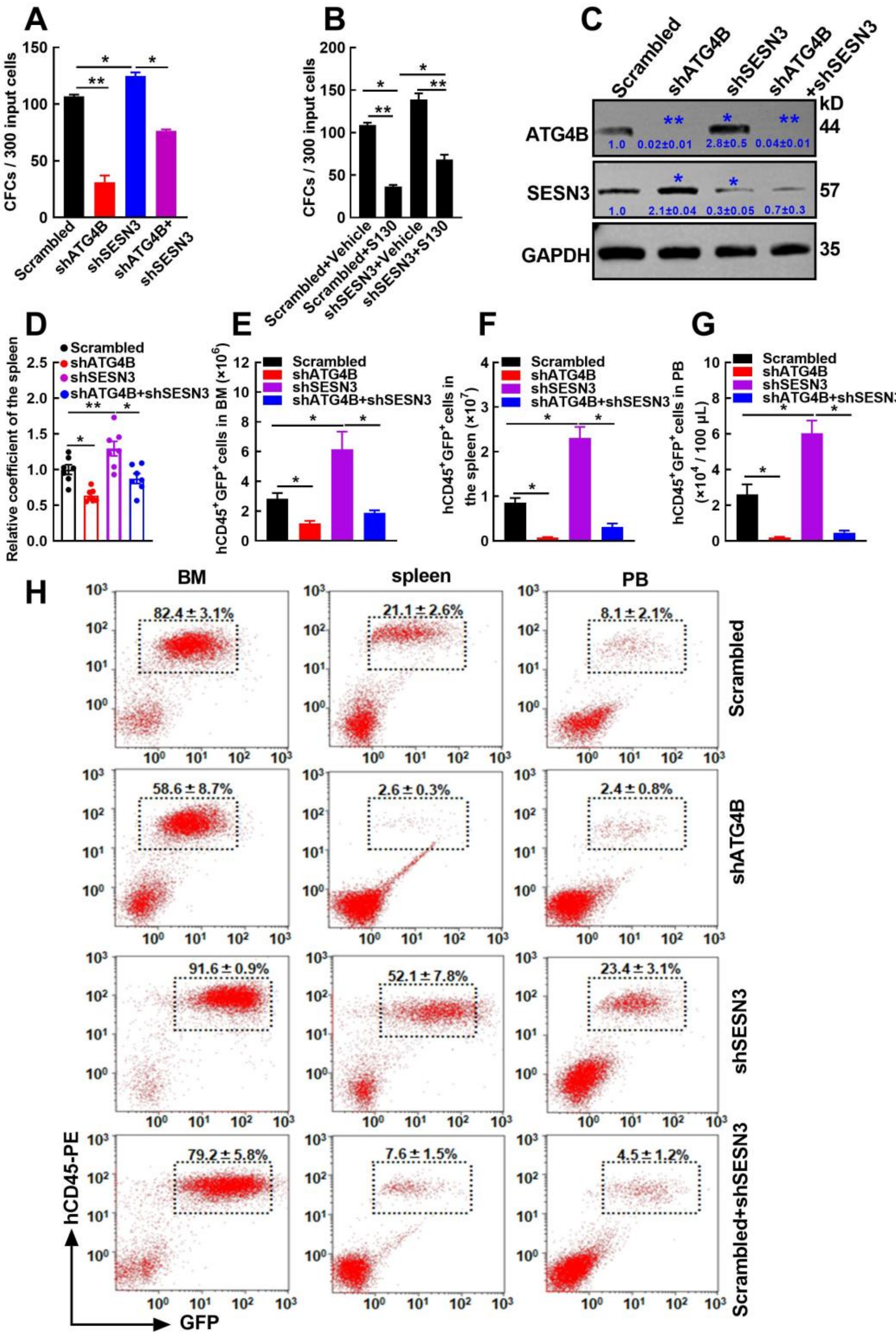

Supplementary Fig. S7 Additional SESN3 silencing reverses the delayed leukemia onset caused by ATG4B silencing. (**A**) Variously transduced Jurkat cells, including control (Scrambled), ATG4B-silenced (shATG4B), SESN3-silenced (shSESN3), and

ATG4B plus SESN3 dually silenced (shATG4B+shSESN3) Jurkat cells were subjected to colony-forming cell (CFC) assay. (**B**) The CFC production in SESN3-silenced and control (Scrambled) Jurkat cells treated with or without S130 was measured (n = 3). (**C**) Variously transduced Jurkat cells were injected into immunodeficient mice, and the leukemic cells in each group of mice were collected for the detection of both ATG4B and SESN3 by Western blotting. (**D**) The relative coefficients of the spleen (ratios of spleen weight to body weight) of these groups of mice were compared. (**E–G**) The absolute numbers of leukemic cells in the bone marrow (BM), spleen, and peripheral blood (PB) of these groups of mice are displayed. (**H**) Representative flow cytometry graphs to analyze the infiltration of leukemic cells in the BM, spleen, and PB are shown. The data are presented as the means ± SEM. Student's *t* test was used to estimate the statistical significance. $^*p < 0.05$; $^{**}p < 0.01$.

Fig. S8

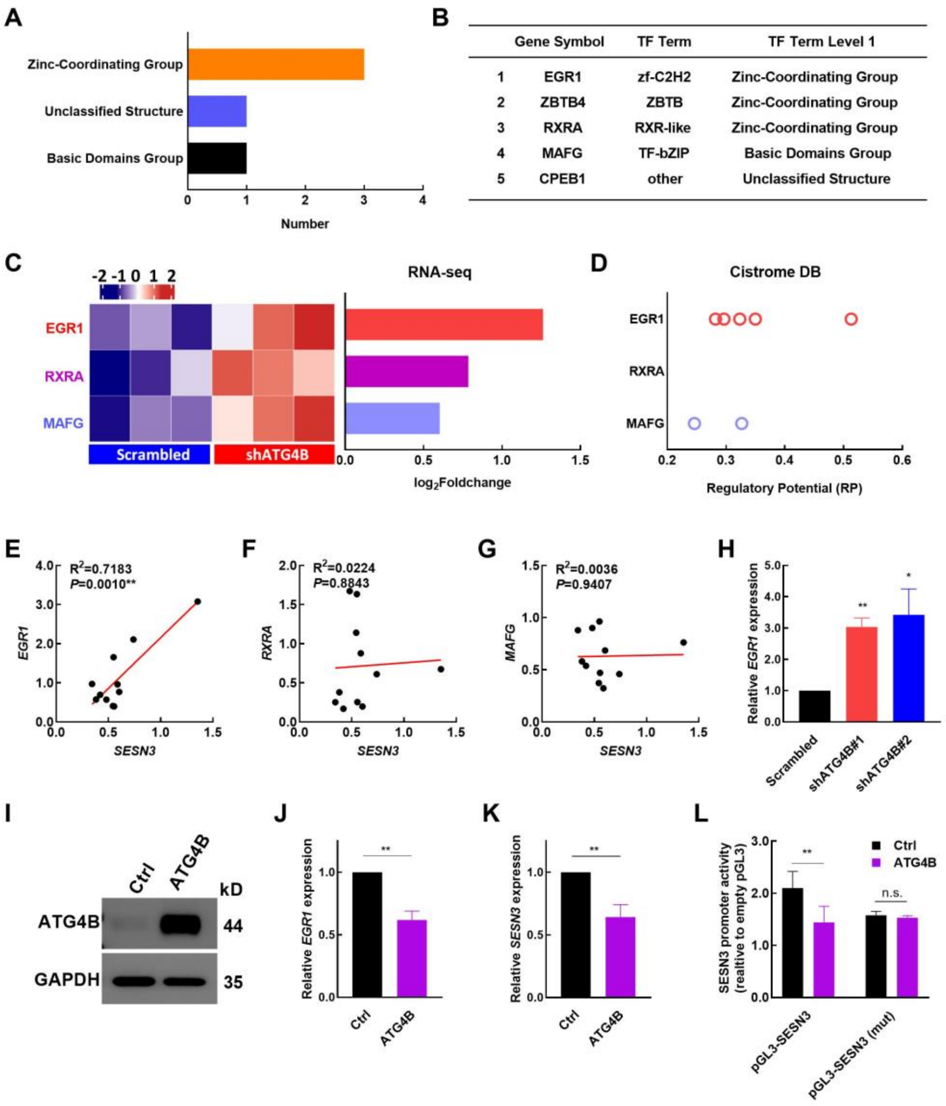

Supplementary Fig. S8 ATG4B regulates SESN3 transcription through EGR1. (**A**) Among the 104 upregulated genes following ATG4B silencing, there are five transcription factors, belonging to three major transcription factor families. (**B**) The list of the five altered genes which encoding transcription factors upon ATG4B silencing. (**C**) The heatmap to show the expression of *EGR1*, *RXRA* and *MAFG* form RNA-seq data. (**D**) The regulation potential analysis of EGR1, RXRA and MAFG on SESN3 using cistrome DB database. (**E–G**) Correlation analysis between *SESN3* and its candidate TF genes (*EGR1*, *RXRA*, and *MAFG*) in primary T-ALL patient samples (GSE7186). (**H**) *EGR1* expression was measured by RT–qPCR in ATG4B-silenced

and control Jurkat cells. (**I**) Western blot confirming ATG4B protein overexpression in Jurkat cells. (**J, K**) RT–qPCR was performed to measure *EGR1* (J) and *SESN3* (K) expression upon ATG4B overexpression in Jurkat cells. (**L**) Reporter assay measuring the activities of wild-type and mutant SESN3 promoters in ATG4B-overexpressed and control Jurkat cells. Data are presented as the means ± SEM. Student's *t* test was used to estimate the statistical significance. *$p < 0.05$; **$p < 0.01$; n.s., not significant.

Fig. S9

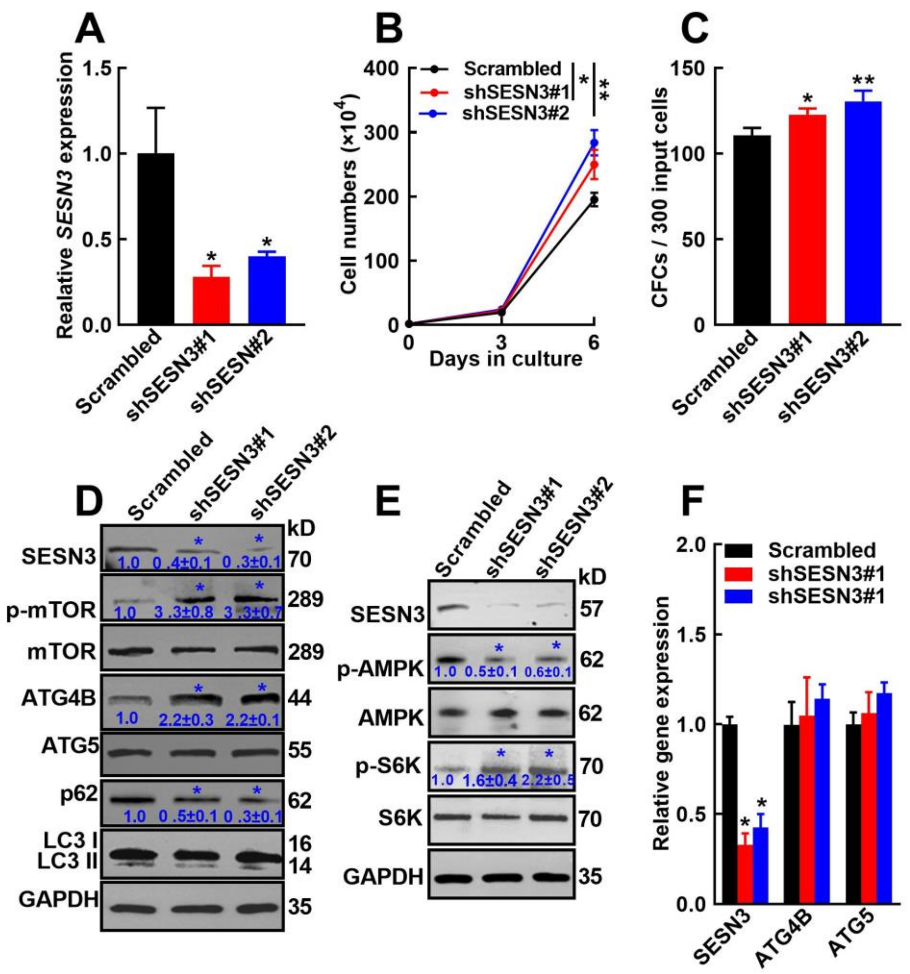

Supplementary Fig. S9 The effects of SESN3 silencing on the growth and signaling in Jurkat cells. (**A**–**C**) Two independent shRNA sequences against SESN3 were delivered to Jurkat cells. The transcript expression of *SESN3* was analyzed by RT–qPCR (A), the growth (B) and colony-forming cell (CFC) production (C) of these cells were analyzed. (**D**) The effects of SESN3 silencing on mTOR, ATG4B, ATG5, p62 and LC3 were analyzed by Western blotting. (**E**) The effect of SESN3 silencing on S6K and AMPK signaling was analyzed. (**F**) The effect of SESN3 silencing on transcript expression of *ATG4B* and *ATG5* was analyzed by RT–qPCR. The data are presented as the means ± SEM. Student's *t* test was used to estimate the statistical significance. *$p < 0.05$; **$p < 0.01$.

Fig. S10

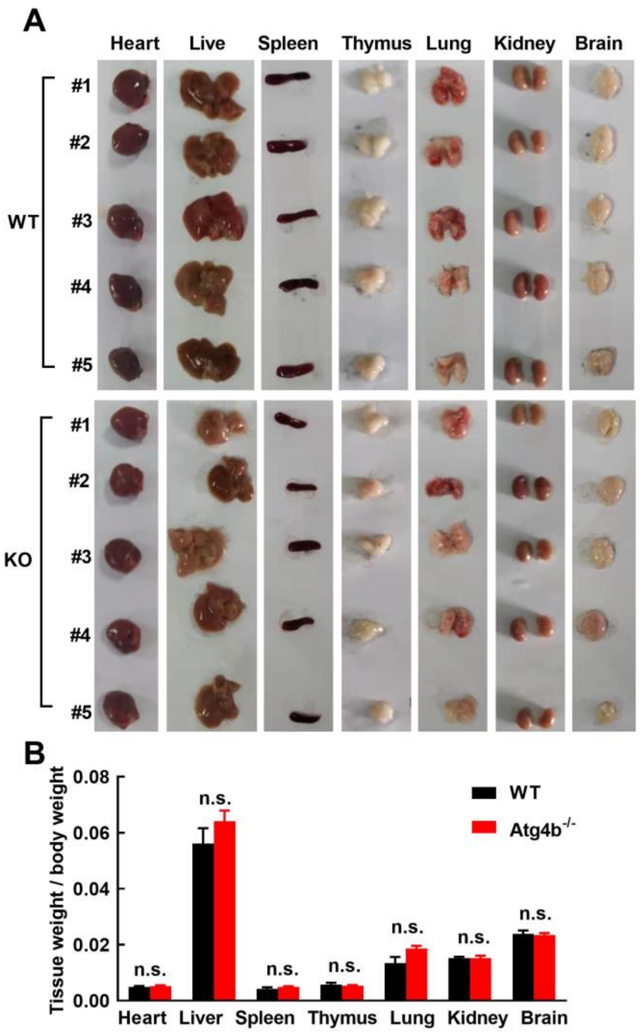

Supplementary Fig. S10 *Atg4b* depletion has no evident effects on the sizes of major organs. (**A**) The representative photos of major organs including the heart, liver, spleen, thymus, lung, kidney, and brain in both WT group and *Atg4b*$^{-/-}$ (knockout, KO) groups of mice are shown (n = 5). (**B**) The effects of Atg4b deficiency on the ratios of major organ weights to body weights were compared (n = 5), including those of the heart, liver, spleen, thymus, lung, kidney, and brain. The data are presented as the means ± SEM. Student's *t* test was used to estimate the statistical significance. n.s., not significant.

Fig. S11

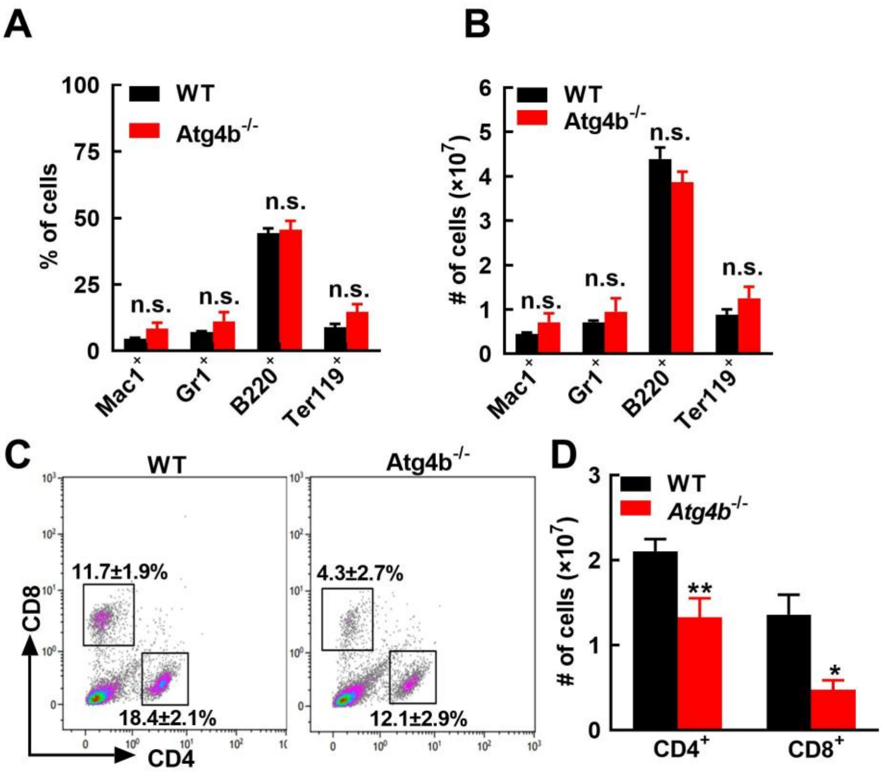

Supplementary Fig. S11 Flow cytometry analysis of hematopoietic cells in the spleen of wild-type and Atg4b deficient mice. (**A, B**) The percentages (A) and absolute numbers (B) of Mac1^{+}, Gr1^{+}, B220^{+}, and Ter119^{+} cells were analyzed by flow cytometry. (n = 5). (**C, D**) The expression of CD4 and CD8 on the surface of spleen cells from WT and *Atg4b*$^{-/-}$ mice was analyzed by flow cytometry (C). The absolute numbers of CD4^{+} and CD8^{+} cells were numerated (D) (n = 5). The data are presented as the means ± SEM. Student's *t* test was used to estimate the statistical significance. $^{*}p < 0.05$; $^{**}p < 0.01$; n.s., not significant.

Fig. S12

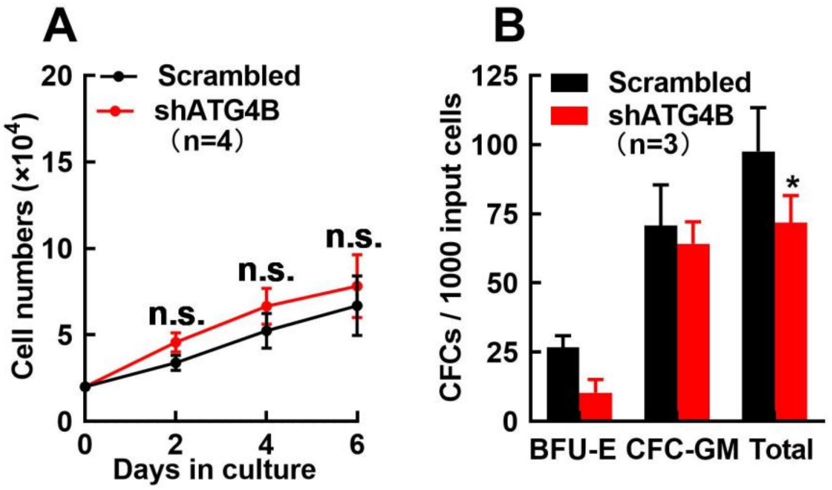

Supplementary Fig. S12 The effect of ATG4B silencing on the proliferation of human $CD3^+$ and $CD34^+$ cells. (**A**) Normal bone marrow (NBM) $CD3^+$ cells (n = 4, A) were activated with CD3/CD28 and cultured with a T cell expansion medium supplemented with IL-2 for two days, then these cells were transduced with lentiviral vectors for the delivery of the Scrambled and shATG4B#1 sequences. Three days later, GFP^+ cells were isolated by FACS, and their growth was analyzed. (**B**) Scrambled and shATG4B#1 were also delivered to NBM $CD34^+$ cells, and the CFC productions of ATG4B-silenced and control cells were compared. BFU-E, erythroid burst-forming unit; CFU-GM, granulocyte-macrophage colony-forming unit. The data are presented as the means ± SEM. Student's *t* test was used to estimate the statistical significance. $*p < 0.05$; n.s., not significant.

Fig. S13

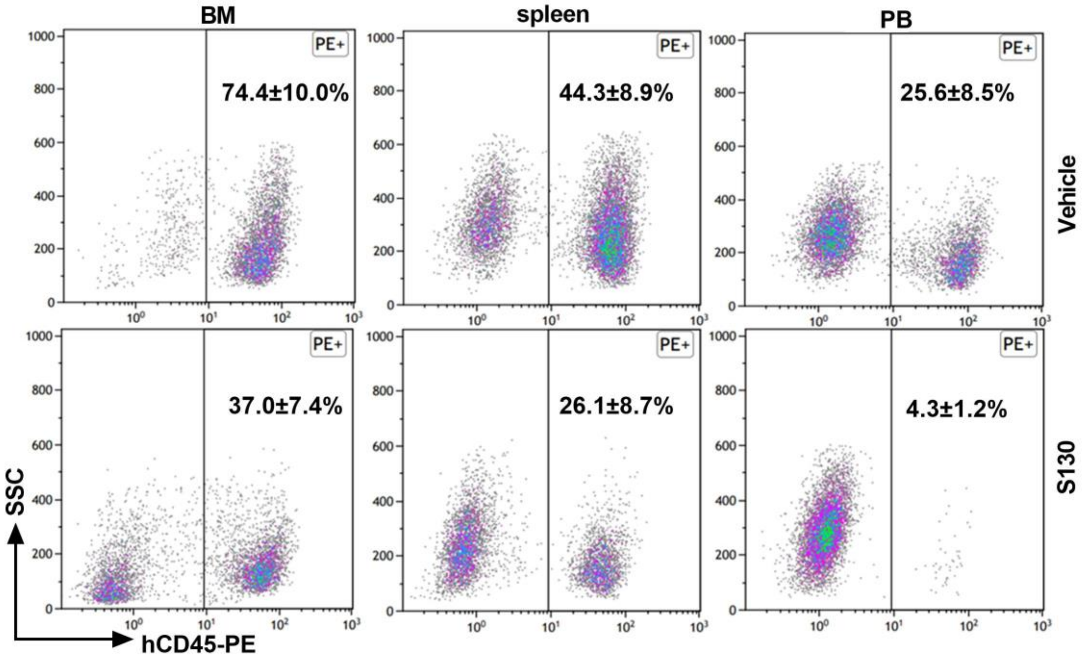

Supplementary Fig. S13 Flow cytometry analysis of leukemic cells in Jurkat cell-induced xenograft model. Jurkat cells were injected into immunodeficient mice through the tail vein, and the mice were allocated into two groups, namely, vehicle and S130-treated groups. The leukemic cells (hCD45^{+}) in the bone marrow (BM), spleen, and peripheral blood (PB) of these groups of mice were analyzed by flow cytometry. The typical graphs of flow cytometry are displayed.

Fig. S14

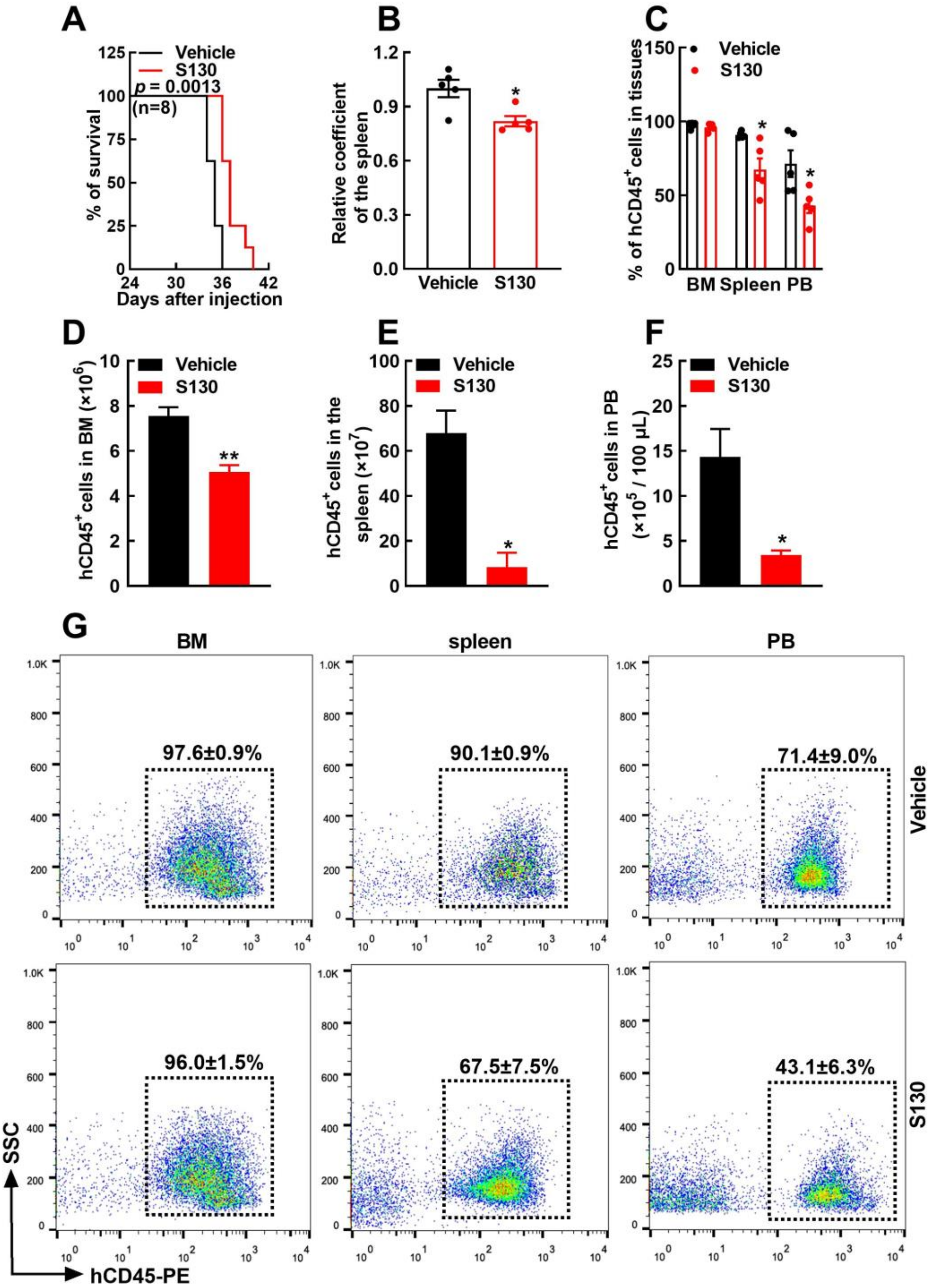

Supplementary Fig. S14 ATG4B inhibitor impairs T-cell leukemogenesis in a patient-derived xenograft (PDX) model. (**A**) PDX cells were injected into immunodeficient mice through the tail vein (1×10^5 cells/mouse and eight mice in each group). Six days after engraftment, S130 (10 mg/kg) or normal saline was administrated to each group for 20 consecutive days. The Kaplan–Meier method was used to study the

survival of these groups of mice, and the *p* value was estimated using the log-rank test. (**B**) The relative coefficients of the spleen (ratios of spleen weight to body weight) in the S130-treated group were compared with those in the vehicle group. (**C**) Leukemic cells ($hCD45^+$) in the bone marrow (BM), spleen, and peripheral (PB) of these groups of mice were analyzed by flow cytometry and summarized statistically. (**D–F**) The absolute numbers of leukemic cells in the BM (D), spleen (E), and PB (F) from these groups are displayed. (**G**) The typical graphs of flow cytometry are displayed. The data are presented as the means ± SEM. Student's *t* test was used to estimate the statistical significance. *$p < 0.05$, **$p < 0.01$.
